# Additive Manufacturing: Unlocking the Evolution of Energy Materials

**DOI:** 10.1002/advs.201700187

**Published:** 2017-07-25

**Authors:** Adilet Zhakeyev, Panfeng Wang, Li Zhang, Wenmiao Shu, Huizhi Wang, Jin Xuan

**Affiliations:** ^1^ School of Engineering and Physical Sciences Heriot‐Watt University Edinburgh EH14 4AS United Kingdom; ^2^ School of Mechanical and Power Engineering East China University of Science and Technology Shanghai 200237 China; ^3^ Department of Biomedical Engineering University of Strathclyde Glasgow G4 0NW United Kingdom

**Keywords:** additive manufacturing, batteries, fuel cells, solar cells, thermal energy

## Abstract

The global energy infrastructure is undergoing a drastic transformation towards renewable energy, posing huge challenges on the energy materials research, development and manufacturing. Additive manufacturing has shown its promise to change the way how future energy system can be designed and delivered. It offers capability in manufacturing complex 3D structures, with near‐complete design freedom and high sustainability due to minimal use of materials and toxic chemicals. Recent literatures have reported that additive manufacturing could unlock the evolution of energy materials and chemistries with unprecedented performance in the way that could never be achieved by conventional manufacturing techniques. This comprehensive review will fill the gap in communicating on recent breakthroughs in additive manufacturing for energy material and device applications. It will underpin the discoveries on what 3D functional energy structures can be created without design constraints, which bespoke energy materials could be additively manufactured with customised solutions, and how the additively manufactured devices could be integrated into energy systems. This review will also highlight emerging and important applications in energy additive manufacturing, including fuel cells, batteries, hydrogen, solar cell as well as carbon capture and storage.

## 1 Introduction

In the 21^st^ century, energy and climate challenges that the world is facing are intertwined. The total global energy consumption is approximately 18 TW,^1^ of which 78.3% was provided by fossil fuels in 2014.^2^ Renewable energy technologies can provide a long term solution for sustainable development, however they cannot replace fossil fuel energy in the short or mid‐term.^3^ Therefore, solutions to increase the efficiency and decrease carbon dioxide emissions from conventional energy conversion processes are necessary. Solar and wind are intermittent energy sources, as a result efficient energy storage processes are required to make renewable technologies more viable. Chemical energy storage offers more flexibility and higher energy densities than mechanical or physical storage.^3^ Generated electricity can be stored in chemical bonds, for example battery charging for mobile applications and production of fuel (hydrocarbons, methanol, hydrogen and ammonia) for medium and large scales.^3^, ^4^ However, the aforementioned energy storage technologies are currently too expensive to enter the market, still require higher performance, as well as the use of environmentally friendly materials.^5^ More importantly, efficient energy conversion, transmission and storage is the ultimate goal in all of the energy sectors.^6^ Advances in energy materials development are crucial to overcome these limitations. The manufacturing methods that can precisely structure such materials in order to fabricate fully functional and efficient energy conversion and storage devices are of paramount importance. Unlike subtractive manufacturing processes, additive manufacturing (AM) can directly produce complex three‐dimensional parts, with near‐complete design freedom, it is advantageous in markets that have a demand for customization, flexibility, design complexity and high transportation costs.^7^ It shows a great deal of potential in manufacturing novel designs of energy conversion and storage devices, which were previously inaccessible via traditional manufacturing methods. Other advantages of AM include reduced lead‐time and it presents itself as a more sustainable manufacturing technology, resulting from less waste material. Purposely this review article will not focus on the detailed information about various AM technologies, since these processes were comprehensively covered in recently published reviews.^8^, ^9^, ^10^, ^11^, ^12^, ^13^, ^14^, ^15^ Instead we will limit this section to a summary of AM processes and will briefly describe how they can collectively aid in resolving energy challenges.

**Figure 1:**
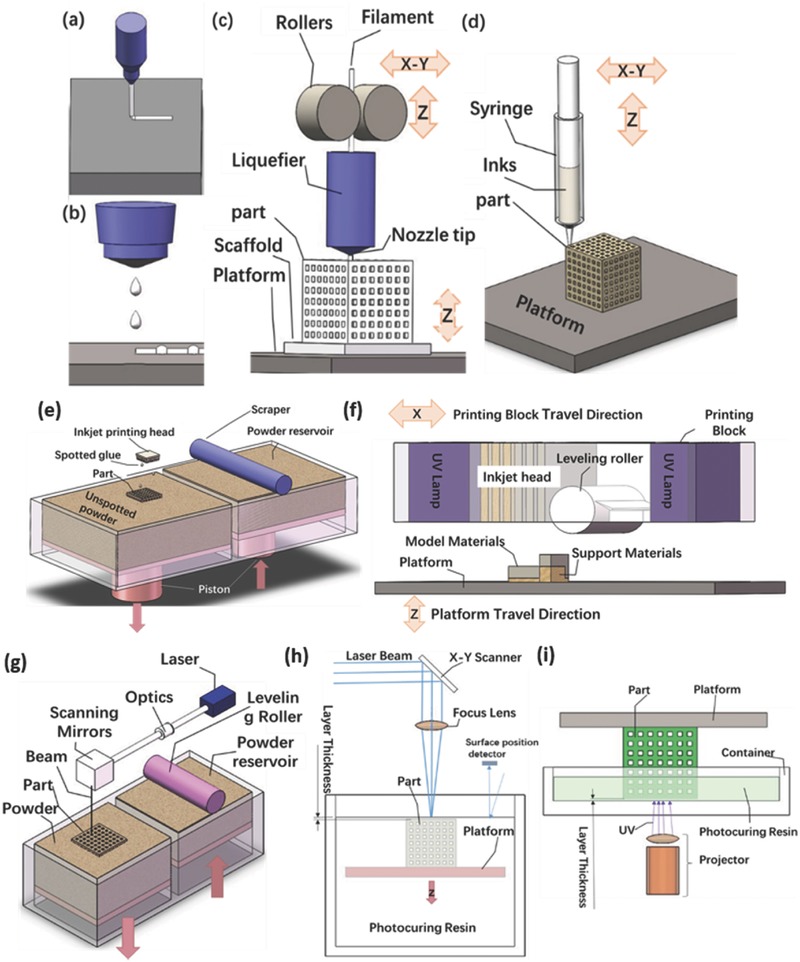
Schematic view of AM methods – a) continuous filament writing and (b) droplet jetting;^23^ c) illustration of the FDM extrusion and deposition process (dashed Z showing the platform travels in Z axis, solid line presenting the nozzle travels);^24^ d) direct ink jet printing;^25^ e) generic illustration of wire form AM method employing metal^26^; f) illustration of the wire fed process of glass;^27^ g) schematic of EHD printing system;^28^ e) the principles of the powder bed inkjet 3D printing;^29^ f) the MJP approach;^20^ g) schematic diagram of SLS process;^30^ h) schematic drawing of free‐surface and (i) constrained‐surface SLA system.^31^

Developed from rapid prototyping, AM, also known as 3D printing, is the umbrella term used to cover a variety of technologies that build structures on a layer‐by‐layer basis, through a series of cross‐sectional slices, that are generated by a computer‐aided design (CAD) software.^16^ According to the American Society for Testing and Materials (ASTM F2792‐12a) there are over 50 different AM technologies, which are classified into 7 different processes: binder jetting, material jetting, material extrusion, vat photopolymerization, powder bed fusion, energy deposition and sheet lamination.^17^ Another simpler way to distinguish these technologies is to group them by the physical state of raw materials, that can be in liquid, solid or powder form; and also by the method used to fuse the material together (thermal, ultra violet (UV)‐light, laser or electron beam).^18^ A number of mature AM technologies (**Figure** **1**), such as fused deposition modeling (FDM), direct ink writing (DIW), selective laser sintering (SLS), stereolithography (SLA), powder bed inkjet 3D printing (inkjet 3D), laminated object manufacturing (LOM)^19^ and their variants (MultiJet printing (MJP)^20^ and electron beam melting (EBM)^21^, ^22^).

It is also worthwhile to mention recent developments in AM that can result in objects with smaller features, faster and cheaper fabrication, as well as the use of materials that previously could not be processed by mature AM technologies. For example, as an alternative to SLS and EBM, metals can also be printed by modified version of FDM and DIW, where laser, electron or plasma beam fuses metal filaments together. A larger build volume can be realized in this approach.^26^ Glass can also be processed in a similar process, where glass filaments are fused together using a laser.^27^ Another novel AM technology, called electrodynamic (EHD) jet printing, is capable of bringing AM closer to the realm of truly micro‐scale fabrication.^28^, ^32^ In this method, deposition of materials is achieved through a nozzle with an inner diameter of 50 µm, which is controlled by a XYZ stage with an accuracy of 100 nm (Figure 1g). Based on SLA, two‐photon polymerization (2PP) is a relatively new AM process that is capable of micro and nanofabrication.^33^, ^34^, ^35^ 2PP involves a photochemical process induced by a femtosecond laser that is tightly focused into the volume of a photosensitive resin (similar to those used in SLA) by a high‐numerical‐aperture‐objective, it differs from SLA by the photoinitiators being able to absorb two photons simultaneously.^35^ 2PP was found to be useful in various fields such as photonic crystals, micromechanical parts, optical and chemical applications.^33^ The accuracy and flexibility of 2PP was also demonstrated in its ability to fabricate nanostructures for bio‐medical applications.^34^ Continuous liquid interface production (CLIP)^36^ is another very recently developed AM technology, which is also based on SLA. CLIP incorporates an oxygen permeable window below the ultraviolet (UV) image projection plane, which creates a “dead zone” (thin uncured liquid interface between the window and cured part), with controlled oxygen inhibition that enables simpler and faster SLA.^36^ Unlike other AM processes, that are very time consuming (due to reliance on layer‐by‐layer printing process) CLIP can fabricate parts at the rate of hundreds of millimeters per hour, which in turn makes it a viable option for mass production.^36^

**Figure 2:**
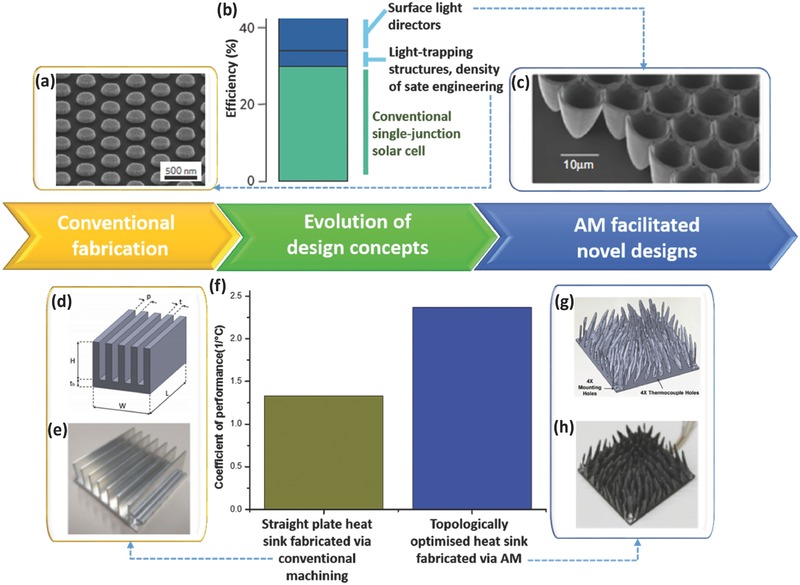
AM for solar energy conversion — a) Hexagonal array of Ag nanoparticles deposited using SCIL. Reproduced with permission.^46^ Copyright 2010, Nature Publishing Group. b) Thermodynamic losses in solar‐energy conversion, with the conventional single‐junction solar cell efficiency indicated in green, entropy losses due to incomplete light trapping and lack of angle restriction indicated in dark blue (left side of the column) and the solutions to reducing entropy losses indicated in light blue (left side of the column). Reproduced with permission.^42^ Copyright 2012, Nature Publishing Group. c) 3D micrometer‐sized parabolic mirror arrays fabricated via 2PP. Reproduced with permission.^43^ Copyright 2013, Nature Publishing Group. AM for thermal energy conversion — d) Schematics of a conventional straight plate heat sink design. Reproduced with permission.^48^ Copyright 2010, the authors. Published under CC‐BY 4.0 license. e) Straight plate heat sink fabricated via conventional machining, f) Performance comparison between straight plate and topologically optimized pin‐fin heat sinks, g) CAD of a topologically optimized pin‐fin heat sink (h) Topologically optimized pin‐fin heat sink fabricated via AM. Reproduced with permission.^47^ Copyright 2015, American Society of Mechanical Engineers ASME.

The commercialization of clean energy systems requires a significant improvement in their performance and energy efficiency. Well‐designed 3D structures were reported to have a potential for increasing the performance of batteries, capacitors, fuel cells and advanced photovoltaic cells,^37^ as well as leading to improvements in reactor engineering and catalysis applications.^38^, ^39^, ^40^ For instance, it was demonstrated that by combining absorbers and reflectors in the absence of sun tracking to fabricate 3D photovoltaic (3DPV) structures, can yield energy densities that are higher by a factor of 2–20 than stationary flat PV panels, compared to the increase by a factor of 1.3–18 for a flat panel with dual‐axis sun tracking.^41^ These self‐supporting 3DPV shapes were achieved by mounting commercial Si cells on AM fabricated 3D plastic frames. AM can also be utilized in fabrication of microstructures that are capable of increasing the efficiency of single‐junction solar cells.^42^, ^43^, ^44^, ^45^ **Figure** **2**b shows that the maximum efficiency realized by the conventional single‐junction solar cell is 28.3% (indicated in green), with dark blue region indicating entropy losses.^42^ One way to increase the efficiency is to fabricate light trapping structures, such as the one shown in Figure 2a, where silver (Ag) nanopatterns were fabricated using substrate conformal imprint lithography (SCIL).^46^ To achieve efficiency beyond the conventional Shockley‐Queisser limit (33% efficiency for a single‐junction solar cell), light directors must be integrated at the surface of a solar cell, to redirect any radiative emission back within the solid angle corresponding to the disk of the Sun, in order to minimize the second entropy loss shown in Figure 2b.^42^ Kosten et al. have recently fabricated 3D micrometer‐sized parabolic mirror arrays, depicted in Figure 2c, via 2PP (previously mentioned AM technology), which have a potential to increase the power conversion efficiency of a single‐junction GaAs solar cell above 38%.^43^ AM's ability to fabricate sophisticated designs, which are otherwise challenging to fabricate using conventional manufacturing techniques, has a potential to allow new design strategies to be introduced in energy applications. For example, Dede et al. used topology optimization with respect to heat transfer and pumping power to design a heat sink for confined jet impingement air cooling (Figure 2g), which was subsequently fabricated out of AlSi12 using AM, as depicted in Figure 2h).^47^ Experimental results showed this novel AM realized heat sink design results in coefficient of performance (COP) being 44% higher than the benchmark straight plate design fabricated via conventional machining (Figure 2d,e). All of the above suggests that AM presents itself as an ideal fabrication method for such complex 3D structures, which are otherwise inaccessible by conventional manufacturing technologies. Multiple hollow parts can be easily 3D printed,^14^ such as enclosed channels, and complex biologically inspired structures were also reported to be fabricated using AM's ability to spatially control local microstructures and chemical composition.^13^

With the global temperatures rising at an unprecedented rate, there is a growing need to accelerate the development of renewable and clean energy from laboratory research to commercialization. Unlike conventional manufacturing techniques, AM offers flexibility of materials that can be processed, as well as reduced time for developing functional prototypes, which provides an opportunity for faster technical development.^49^ On the other hand, in order for the clean energy to reach grid parity, to compete with fossil fuels, the cost of manufacturing of such energy systems needs to be significantly reduced. Not only can AM technology offer rapid prototyping, to receive a tangible feedback during process and product development, but also has a potential for manufacturing of small quantities of customized products at relatively low costs.^7^, ^16^ AM allows fabrication of bespoke products with complex and integrated functional designs in a one‐step process, without incurring any cost penalties, unlike conventional manufacturing technologies, where costs increase with higher design complexity.^7^ Cost savings during production stage can be attributed to reduced handling, shorter supply chains and reduced material use.^18^ Apart from materials, only the 3D model is required to fabricate objects, therefore setup and change‐over costs are negligible, since different CAD files can be uploaded into to an AM machine, without the need in additional tools or molds.^7^ It was reported that AM can result in 40% reduction in waste metals comparing it to machining and subtractive manufacturing.^16^ Currently, AM does not benefit from the economies of scale associated with injection molding and subtractive manufacturing, which currently limits its application to cost‐effective supply of low‐demand custom products.^16^ However, AM is expected to become more cost effective, as the large production becomes more economically feasible.^50^ Since AM consists of a set of several, relatively new technologies, there were few studies that explored its impact on sustainability.^18^, ^50^, ^51^ Generally, AM has several sustainability advantages.^50^

- ability to reduce material waste;
- ability to optimize geometries and produce light weight components, that result in a reduced material consumption during manufacturing and energy consumption during use;
- reduction in transportation, as the parts can be manufactured locally, which can lead to less CO_2_ emissions.

The model developed by Gebler et al. shows that AM has a potential to reduce total energy primary supply (TEPS) by 2.54–9.30 EJ and CO_2_ emissions by 130–525.5 Mt over its entire life‐cycle by 2025.^18^ This implies that AM is starting to transform the manufacturing system and enable improvements in resource efficiency as well as in sustainable production and consumption.^50^

In recent years, there was a growing number of reports on how AM could unlock the evolution of energy materials and chemistries with unprecedented performance in the way that could never be achieved by conventional manufacturing techniques. Having previously discussed how AM can aid the research in energy field, this comprehensive review is intended to fill the gap in communicating on recent breakthroughs in additive manufacturing for energy chemistry and material applications. It will underpin the discoveries on what 3D functional energy structures can be created without design constraints, which bespoke energy materials could be additively manufactured with customized solutions, and how the additively manufactured devices could be integrated into energy systems. This review will also highlight emerging and important applications of AM in energy conversion and storage. Insights into recent developments in 3D printing of functional materials as well as AM of micro and nanostructures and how the integration of these aspects can benefit the energy field will be provided. Finally, reports on recent developments in AM of thermal and chemical conversion devices, fuel cells, batteries, capacitors, solar cells as well as carbon capture and storage devices will be discussed.

## 2 Additive Manufacturing of Energy Materials

**Figure 3:**
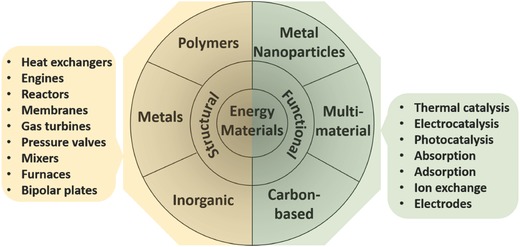
Material that can be processed using AM for energy applications.

The principles of additive manufacturing (AM) have been explained in detail in numerous literatures,^9^, ^29^ but few of them touched on the materials available for various applications, especially in energy field. Fu et al. recently reviewed the research efforts on the use of DIW and development of graphene oxide (GO) based inks to fabricated structures for energy storage, electronic circuits and thermal energy applications.^52^ Consequently, it is necessary to clarify the materials (**Figure** **3**) that can be utilized in AM for the energy conversion and storage applications. There are a few extensive databases on the selection of commercial AM materials available, however Senvol Database, a search engine for industrial AM machines and materials, provides material properties for 981 different ceramic, metal, polymer and composite materials used in commercial AM machines.^53^

### 2.1 Structural Materials for Energy Reactors and Devices

**Figure 4:**
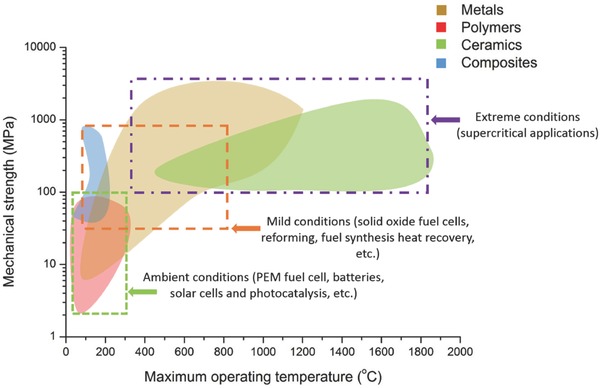
Comparison of structural materials in AM in terms of mechanical strength and maximum operating temperature (information was obtained from references listed in Tables 1, 2, 3.

There is a variety of materials that are utilized in the fabrication of micro and nanostructures in energy applications, but they can be classified into the following categories: ceramics, glass, metals, polymers and composite materials.^29^, ^54^, ^55^ In case of structural parts for an energy device, the compatibility of certain materials, with specific reaction conditions, will depend on various factors, such as strength, conductivity, temperature and corrosion resistance and surface properties. **Figure** **4** is intended to aid in visualization of how mechanical strength and maximum operating temperatures of various groups of structural materials (metals, polymers, ceramics and composites) that can be processed by AM are related to different conditions (ambient, mild and extreme) of energy conversion processes.

#### 2.1.1 Polymers

The low temperature and corrosion resistance, as well las the lack of electrical conductivity limit the use of polymers in energy reactor manufacturing. However, the use of polymers in reactions with mild conditions is very attractive due low cost and ease of handling of polymers compared to metals and other inorganic materials. As the literature^29^, ^56^ shows, thermoplastic polymers, such as polypropylene (PP), polystyrene (PS), polycarbonate (PC), acrylonitrile butadiene styrene (ABS) and polylactic acid (PLA) can be used in FDM, among which the most widely used materials are PLA and ABS.^57^ Moreover, two different materials can be combined into a single filament. In the same way, glass, ceramics and metals can also be incorporated within a polymer. Carbon fibers (CFs) have also been combined with the ABS to extrude filament printed on a FDM 3D printer.^58^ Some of the material properties of polymers and their composites employed in AM are presented in **Table** **1**. Carneior et al.^57^ used polypropylene (PP) and glass reinforced PP (GRPP) pellets as raw material to extrude filaments in a FDM 3D printer to manufacture tensile test specimens for comparison with the same shape objects manufactured by compression molding. The results showed that the printed GRPP samples showed around 30% higher Young's modulus and 40% higher strength values respectively than 3D printed PP, similar to the compression molding samples. Ning et al.^58^ presented another method to improve the mechanical properties of FDM manufactured parts. In this work, the composite filaments were extruded from the extruder after the feedstock – virgin ABS pellets, blended with carbon fiber (3 wt%, 5 wt%, 7.5 wt%, 10 wt%, 15 wt%). Then the specimens used in the following experiment were 3D printed with the use of composite filaments and the pure ABS. The results indicate that adding carbon fibers could enhance the tensile strength and Young's module, and the specimens with 5–7.5 wt% carbon fiber possess the best mechanical properties. Shofner et al.^59^ investigated the mechanical properties of the samples fabricated by FDM 3D printer. Two materials were used as feedstock in this study, one of which is the pure ABS, and the other is a composite material. The composite filaments consisted of the mixture of 10 wt% of vapor grown carbon fiber (VGCF) and ABS, as the matrix. The VGCF reinforced ABS swelled less than the unfilled ABS, and improved the tensile strength, Young's modulus and stiffness over the unfilled ABS by 39%, 60% and 68%, respectively. In the review on AM of composite materials by Kumar et al.^60^ it is reported that although the introduction of fibers can increase the stiffness and decrease the swelling, it can also result in increased brittleness. The addition of linear polymer into the compounds would improve the ductility and flexibility. The effects of addition of metals and ceramics into polymer were also investigated. Composites of nylon 6 and aluminum (Al) and aluminum oxide (Al_2_O_3_) reinforced material, were used to fabricate samples using FDM, and compared with pure ABS specimens for their tribological properties.^61^ This study revealed that the materials combined with Al and Al_2_O_3_ possess better wear resistance, thermal stability and stiffness than the pure ABS material. Masood & Song^62^ developed metal/polymer composite, iron particles in nylon matrix, used for mechanical testing. It was observed that parts consisting of a large amount of iron particles showed lower tensile modulus, tensile stress and elongation, if there is a large particle size distribution. The trend that composites with larger particle size exhibited a higher tensile modulus and stress compared with the smaller ones, was also observed. The thermal and mechanical properties of iron/ABS and copper/ABS composites test samples manufactured via FDM were measured by Nikzad et al.^63^ The metal composites exhibited greater stiffness than the pure ABS.

**Table 1:** Main polymers used in AM

Materials	Printing technology	Tensile strength, MPa	Tensile modulus, MPa	Flexural strength, MPa	Flexural modulus, MPa	Heat deflection temperature, °C	Glass transition temperature, °C; Thermal conductivity, W m^−1^ k^−1^ or Electrical resistivity, Ω cm	Ref.
Polymers								
ABS	FDM, SLA	15–68	1500–4000	48–110	1760–3240	51–99	56–108 °C	56, 58, 63, 64, 65, 66
PP	SLA,FDM, SLS	19–58	585–1660	55–58	1380–1660	51–63	72–74 °C	56, 57, 67, 68, 69
PC	SLA, FDM, SLS	58–68	2690–3100	87–101	2700–3000	48–55	58 °C	56, 70
PA	SLS,FDM	2.4–50	37–4068	37–67	1180–3106	55–182	0.21–0.7 W m^−1^ k^−1^	62, 71, 72, 73
							3.2 × 10^11^–5.9 × 10^13^ Ω cm	
PS	SLS	2.84	1604	N/A	N/A	N/A	89 °C	56, 74
PMMA:	SLA	38–42	1940–2250	73–76	1940–2250	41–48	56 °C	75
Transparent		45–48	1940–2350	81–83	2200–2480	62	N/A	76
Casting		34–37	1000–1600	61–72	1400–2000	41–46	N/A	77
Rigid		0.2–0.4	0.27–0.43	N/A	N/A	N/A	N/A	78
Elastomeric		16–30	701–1700	18–40	N/A	N/A	N/A	79
Engineering Plastics:								
Tough	SLS	37	1517	48	1310	48–188	0.51 W m^−1^ k^−1^	80
Flame retardant		27	1880	41	1462	70–194	1.3 × 10^13^ Ω cm	81
Composites								
ABS‐CF	FDM	44	4018	76	5260	105	N/A	53
ABS‐CNT	FDM	42	2131	80	2174	102.5	105 °C	53
ABS‐Metal	FDM	15	1500–4000	N/A	N/A	N/A	0.2–3.8 W m^−1^ k^−1^	63
PA‐CF	SLS	63–83.4	2900–8900	85–133	3500–7300	173–186	105 °C	53
	FDM	63.9–700	4387–54000	78–470	5650–51000	102–105	N/A	
PA‐Metal	FDM	2.44–3.87	37.18–54.52	N/A	N/A	N/A	N/A	62
PA‐Aramid	FDM	610	27	190	26	105	N/A	53
PA‐Mineral	SLS	51	6.13	76	4.633	183	N/A	53
PA‐Glass	FDM	600	21000	210–420	22000	105–150	0.47 W m^−1^ k^−1^	53
	SLS	27–56.2	2500–7800	37–87.9	2200–4114	157–179	3.2 × 10^11^ Ω cm	73
PAEK‐CF	FDM	106	7.52	172	6.9	N/A	N/A	73
PEEK‐CF	FDM	144	20.6	N/A	N/A	140	143–289 °C	
PLA‐CF	FDM	47.9	4.791	114	6.32	56.2	60 °C	
PETG‐CNT	FDM	N/A	1.794	79	1.779	74	N/A	
PETG‐CF	FDM	55.5	4.93	80	5.74	77	80 °C	
PETG‐Glass	FDM	52	3.285	77.3	3.3	77.3	80 °C	
PEKK‐CF	SLM	110	6.895	N/A	N/A	150	310	

Thermoplastic polymers, such as nylon,^82^ acrylic styrene,^83^ polypropylene^67^ and polyvinyl alcohol,^84^ can also be used in SLS (partial melting method) in trace amounts. They can act as binders along with metals or ceramics; or as the main material, in which the sinter temperature is the melting point of the polymer or one of the materials in a composite.^85^ Wen et al.^86^ developed an approach utilizing SLS to manufacture sand molds for melt casting, to fabricate six cylinder diesel engine head. In this work, Al_2_O_3_ particles, with diameter ranging from 100 µm to 210 µm, were coated with different amounts of binder, which consisted of phenolic resin and hexamethylenetetramine. Powders were irradiated by the CO_2_ laser, followed by baking after the printing of the green parts to increase the strength. The results showed that the printing temperature, post curing temperature and time, the Al_2_O_3_ and binder ratio could significantly affect the strength of the final parts. Shahzad et al.^67^ employed zirconium dioxide (ZrO_2_) and PP, as binder, to print parts in a SLS printer equipped with a 100 W CO_2_ laser, the results showed that the amount of the binder, has a great influence on the strength of the final part. The parts fabricated using composites with 40 vol% PP were very fragile, but 70 vol% increased the strength significantly. Zhu et al.^68^ and Hunt et al.^87^ took advantages of PP and MA956 (an alloy of Fe, Cr, Al, Y_2_O_3_ etc.) to investigate the microstructures and their mechanical properties.

Other AM technologies that involve polymer structure fabrication, such as SLA and inkjet printing, use the advantage of liquid photosensitive resin, as the feedstock.^11^ The resins used in SLA are mainly polyacrylate or epoxy based,^88^ but there are also other resins that can be used in SLA methods, including phenolic resin, etc. In the SLA method, which boasts one of the highest accuracies amongst AM technologies,^88^ the resin used, plays an essential role on the printed model in surface‐free and the constrained‐surface systems.^30^ The exposure of the resin to undesirable ambient conditions can lead to shrinkage, softening or distortion.^89^

**Figure 5:**
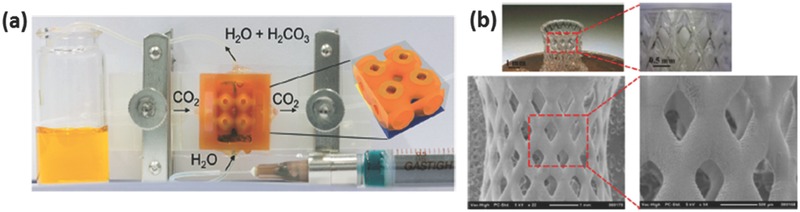
a) Optical image of SLA printed PDMS membrane with effective gas transfer. Reproduced with permission.^90^ Copyright 2014, Royal Society of Chemistry. b) Optical microscopy and SEM images of SLA printed phosphonium polymerized ionic liquids object. Reproduced with permission.^91^ Copyright 2014, American Chemical Society.

In recent years, SLA was employed to 3D print polymeric membrane materials.^90^, ^91^, ^92^ Normally, AM is used to fabricate templates that are then used as molds, onto which polydimethylsiloxane (PDMS) is cast and thermally cross‐linked, however Femmer et al.^90^ were first to demonstrate direct 3D printing of PDMS membranes. They formulated PDMS photoresist, which was then photo‐polymerized using SLA based printer into a 3D gas‐liquid contactor that enabled gas diffusion into liquid phase, as depicted in **Figure** **5**a. SLA was also employed to fabricate 3D phosphonium polymerized ionic liquids objects (Figure 5b), which show excellent thermal and mechanical properties as well as ion conductivity, which exhibits a great potential in ion exchange membrane applications.^91^

#### 2.1.2 Metals

**Figure 6:**
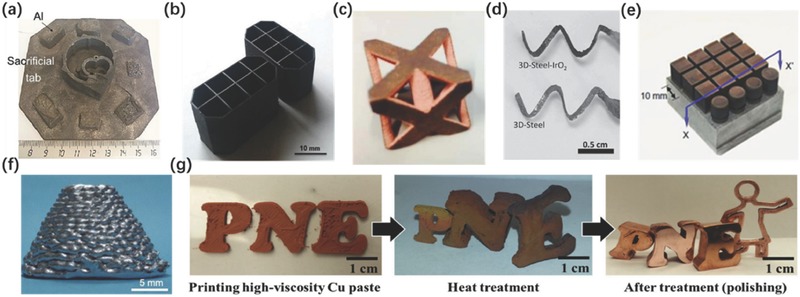
3D printed metal models: a) the CMAs part manufactured by SLS. Reproduced with permission.^98^ Copyright 2014, the authors. Published under CC‐BY‐NC 3.0 license.b) CMAs part manufactured by SLA. Reproduced with permission.^113^ Copyright 2014, Elsevier. c) Copper parts printed by Inkjet printing. Reproduced with permission.^112^ Copyright 2015, Emerald Publishing Group Ltd. d) Photograph of the steel electrode printed and after deposition of IrO_2_ film. Reproduced with permission.^114^ e) SLS cubic electrodes. Reproduced with permission.^97^ Copyright 2014, Emerald Publishing Group Ltd. f) Frustum of a cone structure fabricated by liquid phase. Reproduced with permission.^115^ Copyright 2014, Springer. g) Complete process for fabrication of 3D metal structures from high‐viscosity Cu paste. Reproduced with permission.^111^ Copyright 2015, Springer.

Metals, including stainless steel, copper, aluminum, nickel and other metal composites or alloys are widely used in energy reactor manufacturing due to high temperature resistance, electrical conductivity and ability to withstand high operating pressures. They can also be fabricated using various AM techniques, such as SLS, SLA, inkjet printing and DIW.^29^, ^93^, ^94^, ^95^, ^96^, ^97^ Some of the material properties of metals and alloys used in AM are presented **Table** **2**. Kenzari et al.^98^ used advantages of aluminum based composites, termed as complex metallic alloys (CMAs), as a new material to manufacture structures using SLS approach. CMAs are composed of nylon 12 (PA12) and Al‐Cu‐Fe‐B particles, fused by gas atomization and with a diameter of less than 75 µm. Subsequently the CMAs were employed as the feedstocks printed by SLS methods to fabricate parts shown in **Figure** **6**a, whose mechanical properties after post processing are similar to the printed steel‐brass composites, but also showing lower density by a factor of 2.

**Table 2:** Metals used in AM

Materials	Printing technology	Tensile strength, MPa	Young's modulus, GPa	Yield strength, MPa	Relative density, %	Thermal conductivity, W m^−1^ k^−1^	Max operating temperature, °C	Ref.
Al/Si	SLS, SLA	240–480	N/A	180–270	100	N/A	400	98, 99, 100
Co/Cr	SLS	1200–1260	N/A	850–900	100	N/A	1150	101
NI/Cr	SLS	1350	170	1075	100	11.4	650–980	102
Ti (alloy)	SLS	450–1300	105–120	350–8000	100	6.7–26	425–540	26, 93, 103, 104, 105, 106
Stainless steel: 316L	SLS	500–700	190	300–500	100	15–21	N/A	107
17–4 PH		1100–1300	N/A	620–1100	N/A	N/A	550	108
Maraging steel		1100	N/A	860	N/A	N/A	40 N/A 0	109
Alloy steel		300–600	N/A	N/A	100	N/A		110
Cu	Inkjet 3DP, SLS	116.7	N/A	N/A	85.5	N/A	N/A	111, 112

Aluminum based CMAs sieved with a mesh size of 25 µm can also be mixed with photo‐curable resin containing 30–60 wt% cycloaliphatic epoxy resin, 5–20 wt% aliphatic polyol polyglycidyl and 0.1–10 wt% photo‐initiator and printed in SLA method, which is shown in Figure 6b.^98^ Metallic materials can also be used by inkjet printing (Figure 6c), as Bai et al.^112^ showed by an experiment in which copper particles of various diameters, ranging from 15 to 75 µm, were used as feedstock materials a^113^ long with ExOne's standard binder to fabricate models on a ExOne R2 inkjet 3D. The results show that after sintering at 1080 °C in a tube furnace, a maximum density of 85.5% and purity of 97.3% were achieved by the powder with a 15 µm particle diameter. Ambrosi et al.^114^ used the full melting SLS to manufacture helical electrodes shown in Figure 6d, made of stainless steel. Subsequently these electrodes were employed in different electrochemical applications such as electrochemical capacitors, oxygen evolution reaction, and pH sensing. The results demonstrated that the 3D printed electrodes own excellent capacitive and catalytic properties in alkaline solution. Metals can also be printed in a liquid form as demonstrated by Wang et al.^115^ Metals with a melting point above room temperature and less than 300 °C, including gallium, bismuth and indium based alloys can be adopted as the printing ink and the Bi35In48.6Sn15.9Zn0.4 alloy was selected as printing ink to fabricate models shown in Figure 6f, via DIW method. This experiment demonstrates that 3D metal structures can be rapidly fabricated by this method, due to the high thermal conductivity and capacity of the cooling fluid. However, there are several factors affecting the quality of the print, such as syringe needle diameter, air pressure and the properties of the ink and cooling fluid. Amorim et al.^97^ conducted an experiment involving SLS which utilizes metal composites as a feedstock material to manufacture electrodes. The alloyed Cu‐Ni powders (90 wt% of copper and 10 wt% of nickel) with a particle size of 33 µm were mixed with Mo powder with a diameter range of 3–5 µm to obtain raw materials composed of 63 wt% of Mo. The cubic electrode, shown in Figure 6e, was printed in an EOSINT M250X laser sintering machine equipped with a 200 W CO_2_ laser. The results verified that the smaller layer thicknesses improves bonding, but decreases the porosity of the parts, which was influenced by overlapping. It was also be found that by decreasing the laser scan speed, denser parts could fabricate. Mo‐Cu‐Ni electrodes showed a considerable improvement in performance compared the copper electrodes. Hong et al.^111^ employed the Cu paste as a raw material to fabricate parts depicted in Figure 6g by a screw extruder. In this work, the Cu particles with diameters of 25 µm and 106 µm and polyvinyl carboxy polymer along with poly(vinyl alcohol) were mixed in various proportions to prepare high viscosity fluid. The optimized printing parameters, with the head speed of 6–8 mm s^−1^ and fill density of 75% and 85%, were employed. After sintering, the structure would shrink according to the Cu particles content.

#### 2.1.3 Inorganic Materials

In certain energy applications, inorganic materials present several advantages. For instance, corrosion and temperature resistance of ceramics, which are widely used in solid oxide fuel cells, and the optical transparency of glass, which is beneficial for solar energy applications. Refer to a comprehensive review on AM of ceramic‐based materials by Travitzky et al. for more detailed information.^116^ Some of the material properties of ceramics and glass used in AM are presented in **Table** **3**.

**Table 3:** Ceramic materials used in AM

Material	Printing technology	Tensile strength, MPa	Flexural Strength, MPa	Young's modulus, GPa	Compressive strength, MPa	Thermal endurance, °C	Ref.
Al_2_O_3_	Inkjet, SLA, SLS	260–320	236–430	204–400	2070–2620	1750–1800	73, 95, 117, 118, 119
ZrO_2_	Inkjet, DIW, FDM, SLA	248	600–1500	207	2500	500–2400	73, 116, 120
SiC	Inkjet, SLA, SLS, DIW/FDM	310	324	476	1725–2500	1400	116
SiOC	SLA	N/A	N/A	N/A	10–163	1700	121
Calcium silicate (CaSi)	DIW	N/A	N/A	N/A	48–88	N/A	122

Klein et al.^123^ fabricated a transparent glass model by a new printer based on the DIW technique, in which an annealing chamber was added to the platform and the ink was in liquid glass form, at elevated temperature. In this approach, the commercial soda‐lime glass nuggets were heated up to 1165 °C for 4 h and then fined down for 2 h, within a nozzle at 800 °C to prevent the glass flow. Subsequently, the crucible and nozzle were kept at 1040 °C and 1010 °C respectively, and the platform chamber was kept at 480 °C. The melted glass was then extruded through the nozzle, deposited on the print plate, annealed in the annealing chamber to form the final parts layer‐by‐layer. The printing parameters include melt glass viscosity, temperature distribution in the kiln and layer thickness.

Luo et al.^27^ reported a method to fabricate glass structures by a recently developed AM technology. In this experiment, the researchers used a printer equipped with continuous wave coherent GEM100 CO_2_ laser (λ = 10.6 µm) to fuse glass powders with particle diameters ranging from 1 to 37 µm. The following printing parameters were used: the layer thickness was set at 1 mm and 0.5 mm, laser power at 50 W, scanning speed at 20 mm s^−1^, with beam spot size of 70 µm. In the DIW mode, the laser was set at 25 W and scanning speed at 1 mm s^−1^, meanwhile, the substrate was heated and held at 530 °C to permit a slow cooling speed, resulting in no cracks appearing. This work shows a potential for depositing optically transparent parts by using AM.

Zanchetta et al.^124^ printed ceramics structures using SLA. In this study, 40 g of silicon resin was dissolved in 20 ml of TPM and 20 ml of THF, subsequently, 9.58 ml TMSPM was added into the solution, followed by the stirring for 1 h, and hydrolyzing in acidic condition for 12 h at room temperature. The resulting silicon resin was fed into the CeraFab 7500 printer with lateral resolution of 40 µm and layer thickness of 25 µm, after evaporating at 45 °C for 60 min. Finally, the parts were pyrolyzed at 1000 °C in nitrogen atmosphere with a heating rate of 1 °C min^−1^.

Song et al.^118^ prepared a ceramic slurry composed of grinded alumina, zirconia, PZT and photo‐curable resin, which was milled for 1–2 h at 200 rpm, and exhibited a viscosity of 0.18 Pa s at 30 °C and density of 1.10 g cm^−3^. Thereafter, the resin was utilized in a tape casting integrated SLA 3D printer which is capable of building ceramic parts with high solid content slurries. Then the parts underwent a post processing treatment to obtain full dense ceramic parts.

SLS was also utilized to manufacture ceramic parts, Shahzad et al.^119^ manufactured ceramic structures in two steps. Firstly, green parts were fabricated using polymer/aluminum composites, followed by heat treatment. Raw materials were prepared using following procedures: alumina powders with a mean diameter of 300 nm and PA12 of with a mean diameter of 100 µm were added into the dimethyl sulfoxide (DMSO) with a certain ratio (10 vol%). Then the mixtures were stirred and heated to 140 °C to dissolve the PA, followed by natural cooling, in order to obtain precipitates of Al_2_O_3_/PA composites as a feedstock material. Subsequently the materials were heated to 160 °C on the powder bed, and manufactured on a Sinterstation 2000 machine with a 100 W CO_2_, laser set at 0.176–0.37 J mm^−3^ energy density, in a N_2_ atmosphere. Finally, the parts were sintered to increase the density.

In order to find the suitable parameters of the AM process to fabricate the lithium aluminosilicate glass slurry, Zocca et al.^125^ prepared a LiO_2_‐Al_2_O_3_‐SiO_2_ (LAS) slurry used in a printer with a YAG‐fiber laser. In this work, LAS powders, with particle diameter of several micrometers, are mixed with water and polyethylene glycol (PEG) at 75.5/22/2.5 wt% for a few hours until complete homogenization and stability is achieved. Then the printing parameters: laser power at 15–39 W, scanning velocity at 20–80 mm s^−1^, and layer thickness at 150 µm, were set to determine the optimized parameters. The results show that only at a certain line energy values, the models can be fabricated, which suggests that the line energy (laser power and scan speed) are the main contributors the part's quality.

To solve common problems associated with the traditional ceramic manufacturing methods, AM was introduced by Eckel et al.^121^ for fabrication of ceramic parts. In this study, the expoxy resin was mixed with inorganic backbone consisting of siloxane, silazane or carbosilane, to obtain the pre‐ceramic monomers, which were employed in the next step to print parts in a Formlabs' SLA printer. Subsequently, the parts underwent post‐processing steps to achieve final ceramic parts

### 2.2 Active Materials for Energy Devices

Chemically active materials, such as catalysts, are at the heart of every energy related process; therefore, the choice of an appropriate active functional material is vital to ensure that the reaction occurs at all. Carbon based materials (graphene, graphene oxide (GO), carbon fibers (CFs), carbon nanotubes (CNTs), etc.), nano‐metal particles (NMPs) and composites are generally used as catalysts and supports in energy applications. Active materials also possess other functionalities mentioned in Figure 3, such as adsorption, absorption and ion exchange. Zeolites, metal‐organic frameworks (MOFs), carbon nanotubes (CNTs) and functionalized mesoporous silica are employed for CO_2_ capture, but they can also be utilized as catalyst supports in catalytic conversion of CO_2_.^126^ The morphology of active materials plays an important role, for instance hierarchically structured porous materials can provide large surface area for reaction, interfacial transport and shorten diffusion paths, which is the reason they are widely used in energy conversion and storage systems.^127^ However, the big challenges remain in establishing of a controllable fabrication of structures with fully desired morphology, facets and surface chemistry.^128^ The design flexibility and relatively low cost of AM technologies can aid in the solution of these challenges.

#### 2.2.1 Nano‐Carbon/Graphene Based Materials

Graphene can withstand current densities of up to 4 × 10^7^ A cm^−2^, which is 6 orders of magnitude greater than copper and has a high theoretical surface area of 2360 m^2^ g^−1^, which is similar to activated carbon. It also has outstanding mechanical and optical properties, as a result it is gaining attention of research community in energy storage and conversion applications, such as battery electrodes, supercapacitors, fuel cell catalyst support and solar cells.^129^ Several AM technologies can be used to process carbon based nanomaterials. For instance, it can be a part of a composite material formulation used in SLS or FDM. Alternatively, SLA can be used to fabricate polymeric structures which contain graphene or graphene oxide (GO) within the matrix, which is followed by thermal annealing to burn out polymers and leave a porous graphene or GO structure. GO can also be processed by DIW to directly fabricate graphene based structures, which is described in more detail in the review by Fu et al.^130^

**Figure 7:**
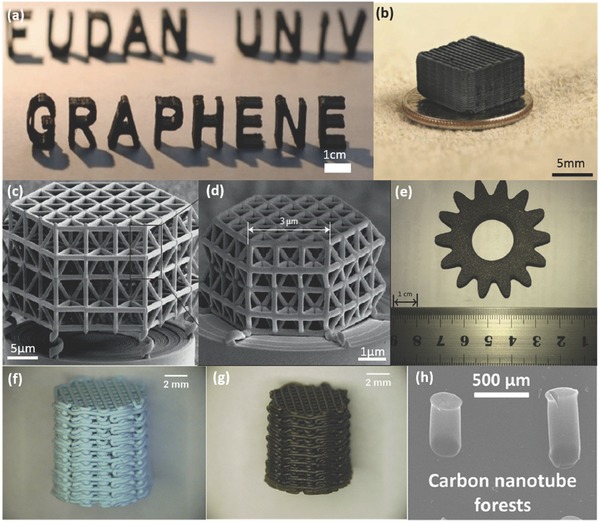
AM fabricated carbon parts. a) 3D printed models fabricated using 3.8 wt% Graphene‐ABS composite filament. Reproduced with permission.^131^ Copyright 2015, the authors. Published under CC‐BY 4.0 license. b) Optical image of the printed microlattices. Reproduced with permission.^132^ Copyright 2015, the authors. Published under CC‐BY 4.0 license.Structures fabricated by 3D‐DLW c) before pyrolysis and (d) after pyrolysis. Reproduced with permission.^133^ Copyright 2016, Nature Publishing Group. e) Parts manufactured by SLS with carbon composites. Reproduced with permission.^134^ Copyright 2016, Elsevier. f, g) Optical images of the Cu/A_l2_O_3_ structures: i) dried, j) sintered. Reproduced with permission.^135^ Copyright 2016, Elsevier. h) SEM image of vertically aligned carbon nanotube forests catalyst sites deposited via inkjet printing. Reproduced with permission.^136^ Copyright 2013, American Chemical Society.

FDM was one of the first methods used to print graphene models which was demonstrated by Wei et al.^131^ In this work, 30 ml of GO‐NMP (N‐Methylpyrolidone) with a density of 5 mg ml^−1^ and 200 ml of ABS‐NMP (15 mg ml^−1^) were mixed together. This was followed by the chemical reduction of GO to graphene sheets with 0.5 ml of 50 wt% hydrazine hydrate at 95 °C. Then the suspension was added to 400 ml of DI water to obtain precipitates. After washing and drying, the composites were extruded from a single screw extruder with a diameter of 1.75 mm. The finished parts, shown in **Figure** **7**a, were fabricated with the prepared graphene filament using FDM 3D printer. Nozzle temperature was set at 230 °C, 80 °C and 130 °C respectively and the layer thickness at 200–400 µm. The G‐PLA was also employed as the raw materials to fabricate filament in this study.

Zhu et al.^132^ illustrated a new approach to fabricate graphene aerogel microlattices shown in Figure 7b. In this experiment, the GO ink was prepared using the following steps: the GO powders with lateral dimensions of 300–700 nm were added to water, by a 20 and 40 mg ml^−1^ concentrations, followed by ultrasonic treatment. Thereafter, the suspensions were mixed with (NH_4_)_2_CO_3_ solution and fumed silica and gelled using organic sol‐gel chemistry to obtain GO inks. Subsequently, GO inks were loaded in a syringe barrel with a tapered nozzle with a 250 µm inner diameter and extruded by air onto a silicon wafers in the isooctane bath. After the fabrication, the structures were cured at 80 °C in sealed glass vials and washed in acetone to remove water. Finally, post processing was conducted to acquire the graphene microlattices, which showed large surface areas, good electrical density, low relative densities and super‐compressibility.

Bauer et al.^133^ employed 2PP to manufacture nanolattice structures shown in Figure 7c,d with strut lengths of 10 µm, 7.5 µm and 5 µm. Additionally, the structures were pyrolyzed at 900 °C in vacuum to get the final nano‐lattices with single struts shorter than 1 µm and diameter of 200 nm, which represents an 80% shrinkage compared to the green parts. The resulting structures showed compressive stresses of 310 MPa at the density of 0.35 g cm^−3^, which six times higher than the micro ones. Moreover, the nano honeycomb structures sustained compressive strengths of 1.2 GPa at 0.6 g cm^−3^, only just lower than the diamond's strength‐to density ratio.

SLS can also be utilized to fabricate carbon structures as Yi et al.^134^ demonstrated. Carbon fibers (CFs) etched by nitric acid (67 wt%) were mixed with phenolic resin 2123 (PF2123) at various ratios in acetone solution, followed by washing and crushing into powders. Thereafter, powders are employed as feedstock material in a SLS printer with a 30 W laser, 280 inch s^−1^ scanning speed and 100 µm scanning space to form green parts (Figure 7e). Finally, the green parts were cured at 180 °C in a chemical vapor infiltration (CVI) treatment for 2 h, and then heated to 1100 °C in natural atmosphere. The obtained parts possessed high density and good mechanical properties with well‐defined features and complex structures. The fact that there was a myriad of pores in the parts and the chemical inertness of carbon made it possible to be utilized as catalyst support.

Jakus et al.^137^ developed 3D printable graphene inks comprised of polylactide‐co‐glycolide (PLG), dichloromethane (DCM), graphene powders, which were thoroughly mixed and sonicated to obtain a viscosity of 30 Pa s. Then the final parts were printed at ambient conditions showing unique mechanical and electrical properties.

#### 2.2.2 Nano‐Metal Based Materials

Adding nanoparticles, including nano‐metals, could improve thermal and electrical conductivity, strengthen mechanical properties, and improve surface finish.^138^ Nanomaterials possess larger surface to volume ratios compared to their bulk counterparts, with higher surface are providing more reactions sites, which leads to an advancements in catalysis of chemical or electric reactions for energy applications. There are generally two main approaches that can introduce nano‐metals into the matrix in AM: 1) printing the structures intermittently, and adding the nanometals into the matrix; 2) pre‐mixing the nano‐metals with the matrix, followed by direct printing.

Crane et al.^135^ used nano‐iron particles with an average size of 7–10 nm to infiltrate the unsupported overhangs printed by inkjet printing using 410 stainless steel and polymer binder as initial materials. After a series of post processing steps such as drying, sintering (1000 °C) and infiltration, the overhangs with nanoparticles reduced shrinkage by 50% and creeping by 95% compared with the untreated one.

Ahn et al.^136^ utilized an omni‐directional printing method, which is based on DIW, and successfully printed suspended structures without supports, planar and flexible microelectrodes, as well as arched architectures. The inks used for printing contained 70–85 wt% of silver nanoparticles with a mean size of 20 ± 5 nm. Annealing was carried out at various temperatures ranging from 150 °C to 550 °C, with the results showing that the electrodes annealed at 250 °C for 30 min can obtain an electrical resistivity of 5.2 × 10^−5^ Ω cm, a value comparable to the bulk silver (10^−6^ Ω cm).

A copper based catalyst was fabricated and utilized in Ullmann reaction by Tubio et al.^139^ In this work, Cu/Al_2_O_3_ inks were prepared and then employed in a DIW syringe to manufacture a woodpile porous structure shown in Figure 7f, possessing high surface to volume ratio and pre‐designed porosity. The inks were prepared by adding 92.31 g of Al_2_O_3_ powders with mean size of 0.5 µm to a 2.56 m aqueous solution of Cu(NO_3_)_2.5_H_2_O, then the solution was modified and concentrated for the extrusion in a 3 ml syringe through a 410 µm nozzle. After drying and sintering (Figure 7g), this structure was employed in several Ullmann reactions. The results indicate that the printed structure exhibited excellent catalytic activity in the reactions without contamination of products, which provided a novel alternative to manufacturing catalytic structures.

#### 2.2.3 Multi‐Material Fabrication

In order to improve utilization of materials in the energy applications and overcome the disadvantages of fabricating structure made of a single material, multi‐material AM techniques can be employed. For instance, Lee et al.^140^ developed alumina based inks using an aqueous colloidal nano‐suspension of aluminum oxide with a mean particle diameter of 100 nm, which were printed by a commercial printer, microdrop Technology GmbH, equipped with a 100 µm nozzle. The inks were printed on microchannels fabricated on the stainless steel substrate. Subsequently, the printed layers were dried and calcined, followed by the impregnation of rhodium from the aqueous Rh(NO_3_) solution. Then microchannels with printed catalyst layer were employed for methane steam reforming, with the inlet concentrations of 10 vol% of CH_4_, 40 vol% of H_2_O, and 50 vol% of N_2_. The results show that the printed layers were thin, uniform, and controllable, and that rhodium can be successfully impregnated into the catalyst layer. The methane conversion of the reaction sustained for more than 60 h, was 98.9% at 700 °C.

Another work involving inkjet printing approach to print catalytic layer had also been conducted by Beard et al.^141^ The suspension of Fe_3_O_4_ in toluene with an average particle size of 10 nm was used as printing ink. Subsequently, catalyst sites were fabricated on the Al_2_O_3_ surface (45 nm), in order to facilitate the formation of nanoparticles after annealing, of a Si wafer with a 100 nm SiO_2_ film on a JetLab 4xl printing system, with a 60 µm diameter nozzle. The printed structures, illustrated in Figure 7h, as well as the carbon nanotubes (CNs), catalyzed during nanoparticle printing and annealed at 800 °C to remove solvents. The results revealed that the growth of the carbon nanotube forests involving in inkjet printing were similar to the chemical vapor deposition (CVD) fabricated ones, but less processing time was required.

### 2.3 Post‐Processing of 3D Printed Materials

Post‐processing steps, a majority of which involve heat treatment, is an important procedure to obtain the final structure for energy applications, especially for the fabrication of metallic,^26^, ^139^ glassy,^27^, ^123^ ceramic,^121^ and carbon^133^ material structures. To remove the binder and acquire the metal structures, the heat treatment is vital. In the work carried out by Hong et al.,^111^ high viscosity copper paste was employed in a FDM printer to manufacture copper structures. After printing, the structures were dried at room temperature in air, followed by sintering at 950 °C for 2 h in the furnace. The heat treatment led to 75% shrinkage of the original volume and void spaces due to the evaporation of the binder. Connections between the metal particles. Bai explored the possibility of using inkjet printing for the fabrication of copper structures, as well as studied the effects of powder size and sintering cycle on the densification of the final parts.^112^ The green parts were heated at 450 °C for 30 min to remove the binder before the following sintering step in air or vacuum at 1000–1090 °C at a heating rate of 5 °C min^−1^ to determine the effects of sintering temperature and holding time on the final density. The results demonstrate that the density of the sintered parts, is proportional to the temperature (1060 °C to 1090 °C), and that sintering can achieve a maximum density of 85.5% of the theoretical value, and sintering in hydrogen atmosphere resulted in density 25.3% higher than after sintering in air. Eckel et al.^121^ reported an approach to manufacture ceramic structures with complex shapes by using preceramic monomers as raw materials in a stereolithography 3D printer. The post processing was conducted following the completion of the printing, in which the green parts were pyrolyzed at 1000 °C at a less than 20 °C min^−1^ heating rate in argon atmosphere, leading to 42% mass loss and 30% linear shrinkage. The obtained SiOC materials exhibited 10 times higher strength than the commercial ceramic foam, and could survive at 1700 °C in air, free from surface oxidation.

Bauer et al.^133^ employed 2PP to fabricate polymeric microstructures with struts of several micrometers in diameter, then the structures were pyrolyzed at 900 °C in a vacuum. After pyrolysis, the structures achieved a shrinkage of 80%, and the struts diameters were shrank to 200 nm. Meanwhile the stresses at failure were reached values of 2–3 GPa, close to the theoretical strength value of glass carbon. The similar work was conducted by Erika et al.,^124^ where after the manufacturing of the ceramic parts, pyrolysis (at 1000 °C in nitrogen with a heating rate of 1 °C min^−1^) was used to obtain the final parts.

However, there are other post‐processing steps which can be combined with the printing system as Wang et al.^115^ and Klein et al.^123^ demonstrated. Wang et al.^115^ used of Bi_35_In_48.6_Sn_15.9_Zn_0.4_ alloy, a low melting point materials, to fabricate structure via modified DIW system. During printing, the syringe needle is immersed into the cooling modules to refrigerate the extruded drops by liquid or air (liquid in this experiment). The subsequent experiment showed that, since the droplets were semi‐solid, it was easy to bond them to the previous layer to form desired structures. Klein et al.^123^ combined an annealing chamber with the DIW system to fabricate optically transparent glass. The annealing chamber was set at 480–515 °C, slightly below the glass transition temperature, to enable improved bonding between layers. The mechanical characterization showed that the adhesion between layers had substantially increased by about 60%, due to the incorporation of the annealing chamber.

Coating is another useful post processing method, Ambrosi et al.^114^ employed SLS to fabricate stainless helical structures for electrochemical applications. In the same work, printed stainless steel electrodes were coated with an IrO_2_ film. To prepare the coating solution, 75 mg of IrCl_4_xH_2_O was dissolved in 50 ml of distilled water, followed by the addition of 180 mg of oxalic acid and 0.45 ml of 35 wt% hydrogen peroxide. Then the pH was modified to 10.5 and the complete conversion was achieved to obtain a stabilized solution. Finally, the printed electrode was immersed into the solution connected to the electrochemical analyzer at a potential scans rate of 0.05 V s^−1^ between −0.6 V and 0.6 V. The helical electrodes before and after coating are illustrated in Figure 6 d.

Some post processing involves multi‐step procedures. To fabricate highly compressible 3D periodic graphene aerogel microlattices, Zhu et al.^132^ printed GO inks on a silicon wafer immersed in isooctane. Thereafter, the printed structure was cured in sealed glass vials at 80 °C for the gelation. Then the washing was conducted in acetone to remove the water in the pores, followed by drying of the supercritical CO_2_ and heating at 1050 °C in nitrogen. Lastly, the HF solution was utilized to etch the silica nanoparticle filler in the structure to acquire the final parts shown in Figure 7b.

## 3 AM of Nano and Microstructures for Energy Application

Devices and materials fabricated at micro and nanoscales poses unique properties of small scale for successful functionality, for instance micro and nanoscale catalysis, combustion, heat and mass transfer is limited by reaction time instead of diffusion time.^142^ The performance of micro energy conversion devices, shows higher power densities and efficiencies than conventional devices.^142^ For instance, majority of hydrogen is industrially produced from natural gas by steam reforming process, which has slow reaction kinetics as well as high capital and operating costs,^143^ whereas micro‐scale reformers possess capabilities that significantly exceed macroscale systems, such as packed‐beds.^144^ Another example of advantages of micro‐energy devices, is catalyst coated microreactor performing with a higher methanol conversion and hydrogen production rate than the conventional packed‐bed reactor.^145^ Microchannel process reactors can greatly increase the efficiency, effectiveness and productivity of chemical and energy production plants, as they are capable of accelerating processes by enabling reactions to occur at rates up to 1000 times faster than conventional systems.^146^ Another attractive feature of micoreactor design is that it can significantly lower capital costs, for instance microfluidic fuel cells can lead to cost reduction by elimination of ion exchange membranes.^147^ Whereas nanostructures can result in significantly larger internal surface area, which enables their application in solar cells, lithium ion batteries, supercapacitors and hydrogen storage systems, all of which involve chemical reactions at a solid‐liquid or solid‐gas interface.^128^ Structural and functional properties of materials may be decoupled via control of the micro and nanostructure, which can be achieved by emerging additive manufacturing techniques.^148^

### 3.1 Screening of AM Technologies for Micro and Nano‐Fabrication

**Figure 8:**
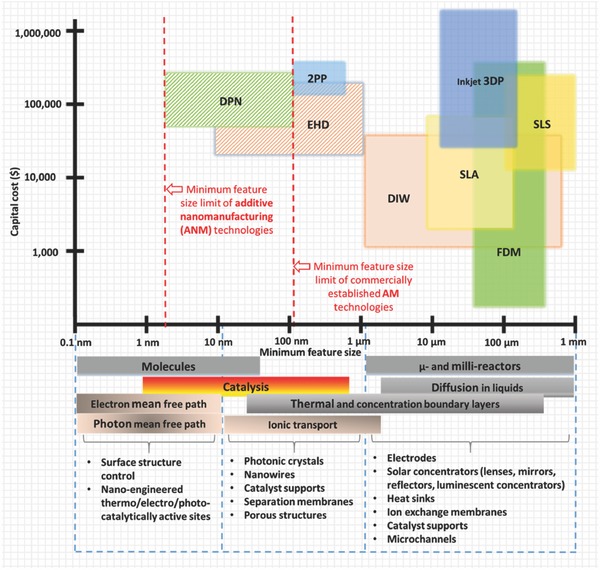
Comparison of AM technologies in terms of achievable minimum feature size and cost:^9^, ^12^, ^23^, ^149^, ^150^, ^151^, ^154^, ^155^, ^156^ transparent squares — commercial AM; shaded squares — novel AM methods capable of nanofabrication that are briefly discussed in section 5.3; length scales of various mesoscale energy processes^49^ and various mesoscale energy structures are listed at the bottom of the diagram.

Various AM technologies differ in the level of resolution they can achieve, printing speed, level of automation, cost and materials that can be printed. Some 3D printing technologies are now capable of fabricating structures with minimum feature sizes ranging from 100 nm to 100 µm.^149^ **Figure** **8** depicts comparison of various commercially established AM technologies (transparent square boxes), as well as novel additive nanomanufacturing (ANM) technologies (shaded square boxes), such as dip‐pen nanolithography (DPN)^150^ and previously mentioned EHD jet printing, in terms of minimum achievable feature size and cost of equipment. Various mesoscale energy processes and their length scales as well as mesoscale energy structures are also included in Figure 8, in order to illustrate the importance of a minimum feature size achievable by a manufacturing technology for energy conversion and storage applications. A comprehensive list of various micro‐AM technologies can be found in the review by Vaezi et al.,^151^ and some of laser based micro‐AM techniques are discussed in more detail in the reviews by Gittard et al.^152^ and Pique et al.,^153^ readers are also referred to the recent review by Engstrom et al.^150^ on ANM technologies.

**Figure 9:**
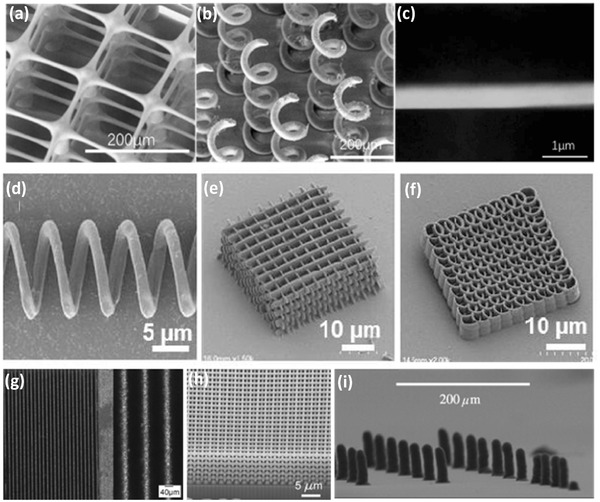
SEM images of 3D microstructures manufactured via μSLA. a) micro matrix with rod suspended beam diameter of 5 µm; b) high aspect ratio 21 × 11 micro rod array, with rod diameter of 30 µm and 1 mm in height; c) the ultra‐fine line with a diameter of 600 nm. Reproduced with permission.^157^ Copyright 2005, Elsevier. SEM images of 3D microstructures manufactured via 2PP – d) microcoil inductor; e) woodpile structure; f) spiral‐like photonic crystal. Reproduced with permission.^158^ Copyright 2016, SPIE. g) LMS fabricated molybdenum walls with 40 µm thickness and high aspect ratio of 1:25. Reproduced with permission.^159^ Copyright 2008, Taylor & Francis. h) Focused ion beam milled cross‐section shown of the calcined 3D periodic TiO_2_ microstructure. Reproduced with permission.^160^ i) EHD printed 3D microstructures. Reproduced with permission.^28^ Copyright 2014, Elsevier.

The freedom of design complexity offered by AM has been widely utilized at the millimeter‐scale, however scaling down to truly micro and nano‐scale structures is only within the reach of some specific AM technologies (SLA, 2PP, SLS and DIW) and require implementation of essential modifications. For instance, micro‐stereolithography (μSLA), utilizes laser spot size with a smaller diameter (few micrometers), compared to commercial SLA technology, which in turn allows 3D printing with 1–10 µm layer thickness.^151^ Sun et al.^157^ successfully developed micro‐stereolithography (μSLA) system that was capable of fabricating complex 3D microstrucutres as illustrated in **Figure** **9**a–c. The precise minimum feature size control is demonstrated by μSLA fabricated suspended beam with a diameter of 600 nm, as show in Figure 9c.

Laser based AM methods, such as SLA, 2PP, SLS are capable of generating complex 3D structures with self‐supporting features at resolutions comparable to or higher than DIW, although their printing speeds and build heights are limited.^149^ The freedom of design (overhangs, enclosed channels etc.) afforded by these laser methods comes at a price, since they require manual post‐printing steps, such as cleaning resin (SLA) or powder (SLS), removing support structures (which is extremely challenging at micro‐scale), whereas extrusion‐based 3D printing (FDM and DIW) are fully automated. Another important consideration is the capital cost of 3D printers. For instance, the expiry of SLA and FDM patents resulted in a number of affordable desktop 3D printers entering the market in the last decade.

The same holds true for SLS, as its patent expired a couple of years ago, a UK based company called Norge Systems launched first low‐cost SLS printer at $13,000 price, which is significantly lower than the $250,000 commercially established SLS systems. Cost is another advantage of DIW technology, as it can be costume built for only $1,000 and have features that are unique for the required applications by the user.

2PP is another technique based on polymerization and currently it is the only AM method able to fabricate arbitrary 3D structures at nano‐scale.^151^ For example, Serbin et al. employed 2PP to fabricate different 3D woodpile structures, that consisted of rods with a thickness of 300 nm, and the in layer rod distance varied between 2 µm and 900 nm.^161^ Recently, in order to expand the functionality of 2PP polymers, researchers have developed multi‐walled carbon nanotube (MWNT)‐thiolacrylate (MTA) composite resins that were used to fabricate electrically conductive 3D micro and nanostructures illustrated in Figure 9d–f.^158^

Commercial SLS printers are not capable of producing microstructures smaller than 500 µm, due to the limited laser spot size, which ranges from 50–300 µm.^151^ Additionally, the powder size and melting process restrict SLS to structures with feature sizes of 100 µm.^23^ Laser micro sintering (LMS)^159^, ^162^ technology was developed in early 2000s and showed a significant improvement in comparison to SLS, with resolution and surface roughness higher by more than one order of magnitude. LMS utilizes sub‐micrometer powders, a cylinder coating blade and a q‐switched solid state laser, which leads to more homogeneous powder coating and higher energy density, allowing LMS to fabricate microstructures with thin 40 µm walls and 1:25 aspect ratios, as shown in Figure 9g.^159^

Direct ink writing (DIW) is limited in the achievable resolution due to the nozzle clogging issues. However, Duos et al.^160^ were able to use direct ink writing (DIW) to print functional oxide sol‐gel inks at microscale. To showcase the capability of DIW, they fabricated a 3D woodpile structure made of titanium dioxide (TiO_2_) as shown in Figure 9h. Rods with thickness of 1.2 µm, which are bonded to one another and span gaps of 4 µm between underlying rods. The authors claim that the broad range of functional materials that can be used by DIW, can find a myriad of potential applications, including energy related ones, such as photocatalysts, photovoltaics and fuel cells. Han et al.^28^ employed EHD to print high aspect ratio 3D structures with sub‐10 µm resolution using phase change ink (wax), as shown in Figure 9i.

### 3.2 Functional AM Fabricated Nano and Microstructures for Energy Applications

#### 3.2.1 Functional Nanostructures

**Figure 10:**
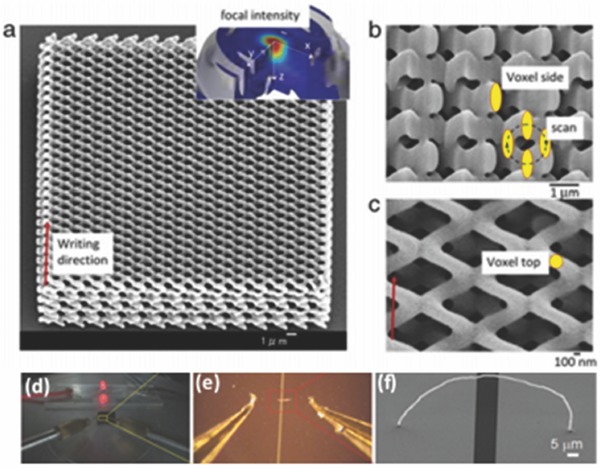
a–c) 3D photonic crystal template fabricated via 2PP. Reproduced with permission.^166^ Copyright 2013, Elsevier. d–f) Optical and FE‐SEM images of rGO nanowire between two gold electrodes and a LED lamp switched on using single rGO nanowire as electrical interconnect. Reproduced with permission.^167^

Photonic crystals can be considered as optical analogous to semi‐conductors, where the periodic dielectric nanostructures of photonic crystals affect photons in a similar way as the periodic potential affects electrons; they also show potential in solar energy related applications.^163^ For instance, 3D photonic crystals can provide a complete photonic band gap, therefore they can be used as emitters for thermal photovoltaic (TPV) power generation.^164^ Since 2PP is capable of fabricating structures with sub‐micrometer features, it was reported to be employed in fabrication of different 3D photonic structures,^165^ such as a photonic crystal template with optical anisotropy,^166^ as illustrated **Figure** **10**a–c.

Kim et al.^167^ were first to demonstrate 3D printed nanostructures composed entirely of graphene. They employed a 3D printing technique based on meniscus guided growth, where a micropipette with 1.3 µm opening was used to form a meniscus of GO ink on a substrate surface, as micropipette was withdrawn, the meniscus stretched out with its cross section decreasing and reaching a nanometer size. As the micropipette was withdrawn, the solvent in GO ink evaporated rapidly, forming a freestanding GO nanowire, which was subsequently reduced forming rGO nanowire with a radius of 400 nm, as shown in Figure 10d–f. They were able to fabricate rGO nanowires with 100 nm radius and demonstrated the use of 3D printed nanowires as electrical interconnects, as well as 3D transducers in CO_2_ gas sensors.

#### 3.2.2 Functional Microstructures

**Figure 11:**
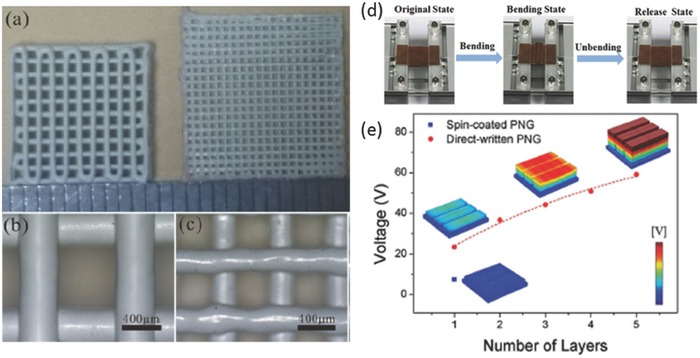
Optical images of a DIW fabricated piezoelectric woodpile stuctures — a) top of 2 samples with different samples and magnified view of (b) 10 mm x 10 mm sample with 400 µm filament diameter and (c) 12 mm x 12 mm sample with 250 µm filament diameter. Reproduced with permission.^168^ Copyright 2015, the authors. Published under CC‐BY 4.0 license. d) Optical images which illustrate the mechanism of electricity generation by DIW fabricated PNG device in its original, bended and released state and (e) simulation results of voltage output of spin‐coated PNG and DIW fabricated PNGs with different number of layers. Reproduced with permission.^169^

Li et al. prepared piezoelectric ink with Li, Ta, Sb and co‐doped (Na,K)NbO_3_ (KNN) powders to fabricate 3D piezoelectric scaffolds (**Figure** **11**a–c) with diameters at micrometer scale by using DIW.^168^ Recently Gao et al.^169^ also employed DIW, with an ink containing KNN nanowires and PDMS, to fabricate flexible KNN nanowires based piezoelectric nanogenerator (PNG), as shown in Figure 11d. DIW allowed for simultaneous micropatterning of structure as well as alignment of KNN nanowires on a bottom electrode, which resulted in DIW printed PNG's output voltage (21 V) being 400% compared with that of conventionally spin‐coated one (5 V), as illustrated in Figure 11e. They demonstrated that the DIW fabricated PNG device can successfully harvest mechanical energy and has a great potential in self‐powered sensors as well as in portable and wearable electronics.

**Figure 12:**
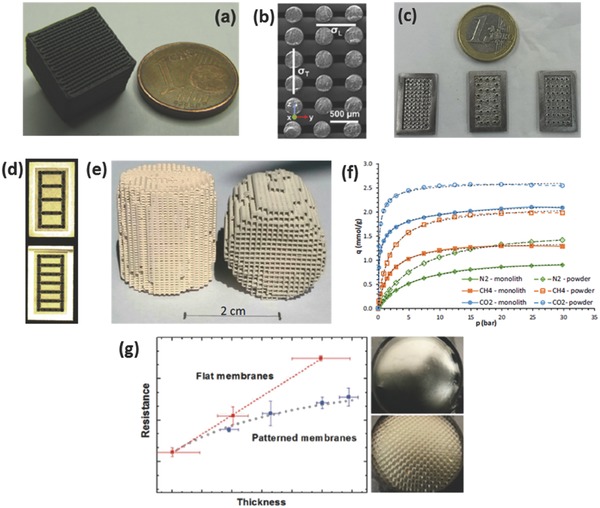
a) Optical and (b) FESEM images of 10 vol% GNP/SiC DIW printed and sintered scaffolds. Reproduced with permission.^170^ Copyright 2016, Elsevier. c) Optical image of different micro fuel cell flowfield plate designs and (d) x‐ray image of enclosed channel micro fuel cell flowfield plates. Reproduced with permission.^171^ Copyright 2016, Elsevier. e) Optical image of ZSM‐5 zeolite monolith with 400 µm fiber diameter and (f) adsorption isotherms of CO_2_, CH_4_, N_2_ on H‐ZSM‐5 powder and monolith. Reproduced with permission.^172^ Copyright 2017, Elsevier. g) photomicrographs of flat and micropatterned membranes and their comparison in terms of ionic resistance. Reproduced with permission.^92^ Copyright 2016, American Chemical Society.

Roman‐Manso et al.^170^ demonstrated a first example of using 3D printing to fabricate graphene/ceramic composite structures with an aim to provide graphene network with mechanical stability and resistance to abrasion, wear and corrosion to most chemicals. They employed DIW, with a nozzle diameter of 300 µm, to fabricate graphene/silicon carbide (SiC) lattice microstructure (**Figure** **12**a–b), which was subsequently consolidated by Spark Plasma Sintering (SPS). The electrical conductivity (σ) of these scaffolds was estimated to be 611 S m^−1^ in longitudinal (σ_L_) an 273 S m^−1^ in transverse (σ_T_) orientations relative to the extruded rods. The authors envision that these 3D printed graphene/SiC structures can be used for energy storage/conversion, gas sensing and catalyst support applications.

AM has also found use in ultrafast prototyping of micro fuel cell flowfield plate designs.^171^, ^173^ For instance, SLM was used to fabricate and compare different flowfield plate designs, shown in Figure 12c,d, for micro fuel cells that use hydrogen and oxygen as reactants.^171^ The experimental results show that stainless steel micro fuel cell flowfield plates with enclosed channels showed higher power (363 mW cm^−2^) and current (1.515 A cm^−2^) densities compared to a traditional open groove counterpart.

Another important component of fuel cells is an ion exchange membrane. Recently, Seo et al.^92^ 3D printed a micropatterned anion exchange membrane, consisting of diurethane dimethacrylate (DUDA), poly(ethylene glycol) diacrylate (PEGDA), dipentaerythritol penta‐/hexa‐acrylate, and 4‐vinylbenzyl chloride (VBC), by employing SLA based process. The performance of 3D printed poly(DUDA‐co‐PEGDA‐co‐VBC) anion exchange membrane with micropatterns (raised features with heights varied from 190 to 589 µm) was compared to its 3D printed flat counterpart, as shown in Figure 12g. The experimental results showed that micropatterned membrane had lower ionic resistance than flat membranes, when the effective thickness, which is volume/area ratio, was kept the same for both samples.

Apart from energy conversion and storage applications AM is also capable of fabricating monoliths, with microscale features, for carbon dioxide capture (CO_2_). For instance, Couck et al.^172^ employed DIW based 3D printing method to construct zeolitic monolith with inter‐connected channels (Figure 12e), composed of 65 wt% ZSM‐5 zeolite and 35 wt% of binary binder (50 wt% bentonite and 50 wt% ludox), with fiber thickness of 400 µm and porosity of 68% for separation of CO_2_ from methane (CH_4_) and nitrogen (N_2_). The experimental results showed excellent separation performance of 3D printed ZSM‐5 monolith, with CO_2_ being the most strongly adsorbing component. Comparing the performance of 3D printed ZSM‐5 monolith with its powder counterpart, the adsorption capacity of the monolith decreased slightly, as shown in Figure 12f, this can be explained by the presence of the binder (35 wt%).

#### 3.2.3 Functional Multi‐Scale Structures

**Figure 13:**
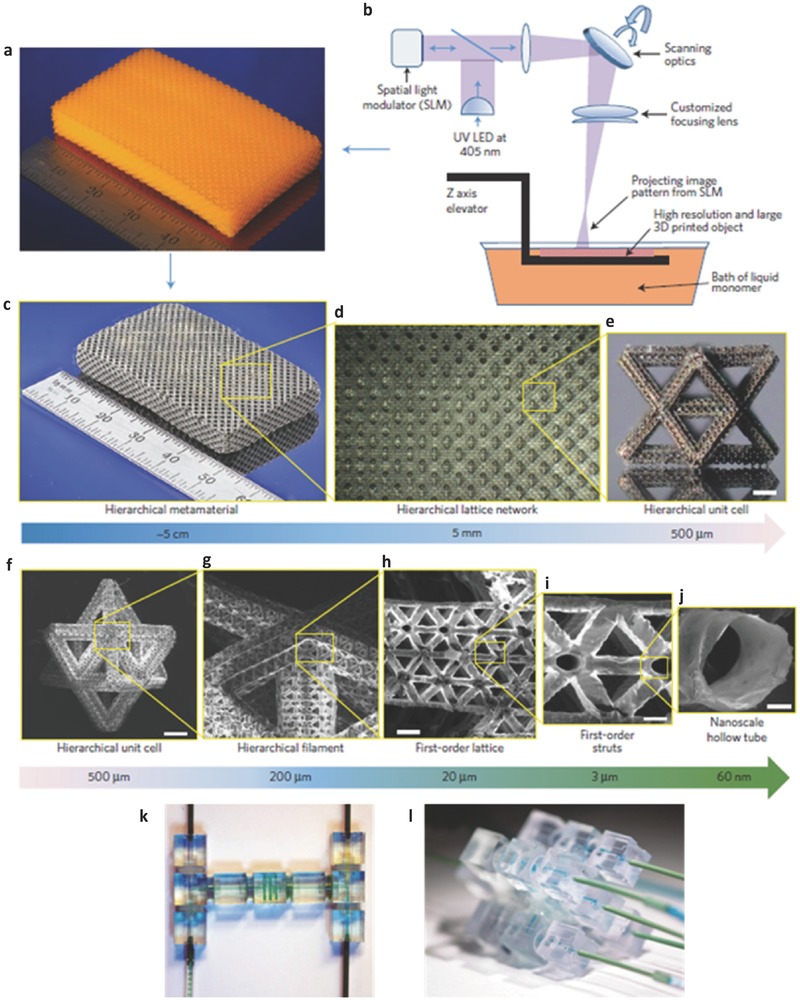
a) LAPμSLA printed polymer metamaterial template, b) LAPμSLA – additive manufacturing technique for fabrication of hierarchical metamaterials and c–j) optical microscope and SEM images of hierarchical lattice material with a network of hierarchal stretch‐dominated octet unit cells and its multi‐scale features. Reproduced with permission.^174^ Copyright 2016, Nature Publishing Group. k) the printed T‐junction device mixing of the two water flow bearing different dyes and (l) the reconfiguration of the printed modules. Reproduced with permission.^175^

As demonstrated in previous sections, 3D micro and nanostructures show a great deal of potential in energy field. In order to take advantage of these architectures and bring them from laboratory scale to full commercialization, there is a growing need for multi‐scale fabrication methods. Zheng et al.^174^ were able to fabricate metallic metamaterials that contain hierarchical 3D topologies with feature sizes ranging over seven orders of magnitude in length scale, from tens of centimeters to tens of nanometers, as shown in **Figure** **13**a–j. This was enabled by a scalable AM technique called Large Area Projection Microstereolithography (LAPμSLA), schematics of which are shown in Figure 13b, which was used to build a polymer hierarchal multi‐scale structure template (Figure 13a). After electroless nickel deposition on template, polymer material was chemically removed, producing hollow tubes with the thicknesses reaching 50 nm, thus creating a metallic structure with smallest features in nanoscale in a multi‐centimeter sized object. The resulting multi‐scale structure span over 7 orders of magnitude and the entire object showed high tensile elasticity (more than 20%), low relative density (below 0.1%) and specific tensile strength of 40 MPa g^−1^ cm^−1^.

Another way of fabricating devices with macroscale overall dimensions and microscale functional features can be achieved by 3D printing microscale components with connectors that can be used to assemble them together. Bhargava et al.^175^ utilized this approach using SLA, which involved manufacturing microfluidic component designs based on discrete elements, which can be easily assembled into complex, modular and robust 3D structures according to reaction requirements. One such example is a reconfigurable microdroplet generator, developed in this work. A T‐junction configuration, shown in Figure 13k, was employed to generate droplets, and the two dye bearing mixed aqueous streams were driven by a pump, with a highest flow rate of 2.5 ml h^−1^, sheared by the carrier oil phase at a rate of 1 ml h^−1^, resulting in a steady state submillimeter droplet formation. In addition, the T‐junction device can also be converted into a 3D structure by regrouping the elements for a high throughput operation, shown in Figure 13 l. This particular design was adapted to facilitate intricate operations, such as handling multiple fluid streams, which proved to be challenging in the planar configurations. Since reactor components, can be readily assembled according to requirements of different reactions within the structure, this method offers advantages of ease of system maintenance and low cost. The aforementioned novel 3D structures show a tremendous potential in energy applications, due to decreased pressure drop, more homogeneous distribution, as well as faster and uniform mixing compared with the traditional planar structures. However, the complexity of fabrication, packaging issues and high manufacturing costs are barriers to large scale power applications of micro/nano energy conversion devices.^142^

## 4 AM for Energy, the Applications

Chemical and geometrical control at multiple length scales is of great importance in the design of energy systems. In recent years, there was a rapid acceleration of scientific progress in nanoscale design for renewable energy applications.^176^ This trend is understandable, since a number of vital physiochemical interactions, as well as catalytic, electronic and optical properties are all affected by ability to tune the building blocks at the molecular and supermolecular scale.^40^ At the same time, many chemical, mechanical and optical properties are also affected by features above nanoscale, therefore the link between nano‐, micro‐ and macroscales is very important in the design of efficient energy conversion and storage systems. Even though the majority of established AM technologies cannot reach the realm of nanofabrication, the advantages offered by AM over conventional manufacturing methods, as well as ability to fabricate active materials and functional microstructures, show a great deal of potential in in certain energy applications. The utilization of unique properties of nanomaterials, requires their programmed assembly into higher hierarchical structures.^177^ Moreover, combining recent advances in nanomaterials research with AM's ability to fabricate multiscale structures can result in fully functional energy devices. This section is intended to highlight emerging and important applications of AM in fabrication of energy conversion and storage devices, including fuel cells, batteries, hydrogen, solar cells, photocatalysis as well as carbon capture.

### 4.1 Chemical Reactors for Energy and Fuel Processing

**Figure 14:**
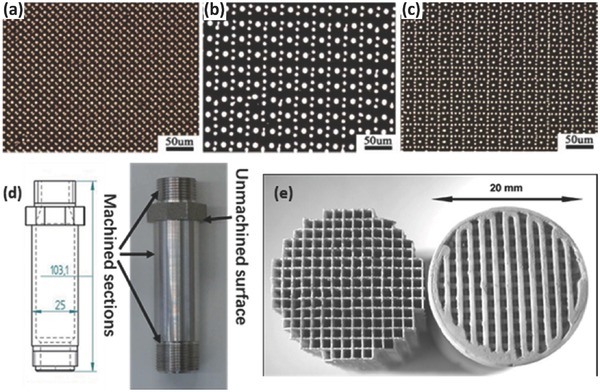
a–c) Optical micrographs of different catalytic patterns. Reproduced with permission.^179^ Copyright 2014, Elsevier. d) Blueprint and AM fabricated reactor after machining. Reproduced with permission.^182^ Copyright 2017, Royal Society of Chemistry. e) Monoliths with similar outer dimensions and surface areas coated with barium manganese hexaaluminate: honeycomb fabricated via conventional extrusion (left) and lattice via DIW (right).. Reproduced with permission^181^

Ability to control the microstructure and geometry of active materials, not only provides increased surface to volume ratios, but at the same time requires less catalyst loading, which can result in process intensification and cost savings. However, not all AM technologies are capable of directly processing active materials into catalytic microstructures. One approach to decrease mass transport limitations in heterogeneous catalytic reactions, is to employ hierarchically structured porous materials, with an optimized network of macropores and mesopores.^39^ AM can be easily used to fabricate structures with micrometer features and overall macroscale dimensions, where the geometry, channel diameter, porosity can be controlled to fabricate catalytic supports, and they can be subsequently coated with a mesoporous catalyst layer. This in turn, can result in high surface to volume ratios and enhanced mass and heat transfer, leading to active and stable 3D microstructured reactors.^178^ Luo et al.^179^ used inkjet printing to fabricate nano‐Pt catalytic patterns used as micro‐heaters, in micro‐electronmechanical systems. The printing inks were prepared by dissolving chloroplatinic acid powder in water at 0.01 mol L^−1^, then waiting for 48 hr and filtering to avoid nozzle clogging. Thereafter, the prepared inks were introduced into the Autodrop micro dispensing system equipped with a nozzle with a 70 µm inner diameter, to print various patterns, shown in **Figure** **14**a–c, at a droplet flight speed of 1 m s^−1^. The printed structures were utilized to catalyze methanol combustion. The results suggest that the methanol conversion rate of the inkjet printed structures was almost doubled when compared with the samples fabricated by hand painting. Finally, the ultra‐low loading, 0.014 mg cm^−2^ and high utilization of 34710 mW mg^−1^ of Pt catalyst were achieved by inkjet printing approach. A further investigation was conducted by the same group to determine the optimized printing parameters and acquire the uniform deposition in order to avoid the coffee ring effect.^180^ In this study, the optimized inkjet parameters were obtained and ethylene glycol (EG) was added into the solution to achieve the uniform deposition. Subsequently, the structures were heated to get pure platinum for the methanol/air oxidation reaction. Stuecker et al. employed DIW to fabricate catalytic ceramic support structure, shown on the right hand side of Figure 14e, in order to enhance the catalytic combustion of methane gas.^181^ Experimental results showed that DIW fabricated lattice converted 6 times more methane at 600 °C than conventionally extruded‐honeycomb support, shown on the left hand side of Figure 14e. The authors attribute this significant increase in catalytic combustion of methane to the fact that in DIW fabricated lattice the rods, with diameters ranging from 725 µm to 1 mm, are oriented in such a way to create ‘no line‐of‐sight pathways’, to have high surface to volume ratios (15–44 cm^2^ cm^−3^) and to generate highly turbulent flow promoting increased mass transfer, without sacrificing low pressure drops when compared to conventional extruded‐honeycomb support.

More recently, Peters et al.^182^ employed selective electron beam melting (SEBM) to fabricate a steel reactor for the dehydrogenation reaction. In this study, Ti‐6Al‐4V powders with a particle size ranging from 15 to 105 µm were sintered by an Arcam S12 system in He atmosphere, with the layer thickness set at 100 µm. The fabricated reactor with cubic diamond cellular structure, as shown in Figure 14d, was subsequently trimmed to allow for sealing. Then the commercial boehmite powder and disperal were dispersed and stirred in diluted nitric acid solution (pH = 2), thereafter, the catalyst powder, 5% Pt/Alumina, was added into the former solution to achieve a suspension containing 7.5 wt% AlO(OH) and 7.5 wt% Pt/Alumina after 1 h of stirring. The coating was conducted by filling the chamber with the suspension, then dried and calcined to obtain a coating mass of 3.5 g per reactor. The reactor was mounted in a ventilated oven, and H12‐NEC (perhydro‐N‐ethylcarbazole) was introduced at a rate of 0.5–4 ml min^−1^ at a temperature of 220–260 °C, to carry out the dehydrogenation reaction. Finally, ten reactors were scaled up to form hydrogen release unit (HRU). The hydrogen generation of H_2_ was 9.8 NL min^−1^ in a catalytic reactor with a total volume of 250 mL.

**Figure 15:**
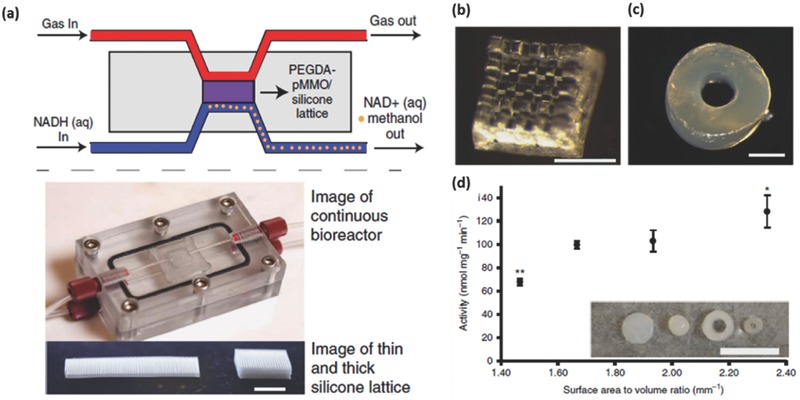
a) Schematics and image of the flow‐through bioreactor and 2 silicon lattice structures used to support PEG‐pMMO hydrogel membrane (scale bar 1 cm); b) P*µ*SL printed PEG‐pMMO grid structure with small feature size (scale bar, 500 µm); c) P*µ*SLA printed PEG‐pMMO cylinders with varying surface area to volume ratios on a shorter timescale with reduced resolution (scale bar, 1mm); d) The dependence of PEG‐pMMO activity on surface area to volume ratio, insert: printed cylinders with surface to volume ratios of 1.47, 1.67, 1.93 and 2.33 (scale bar, 10 mm). Reproduced with permission.^183^ Copyright 2016, the authors. Published under CC‐BY 4.0 license.

Blanchette et al.^183^ developed biocatalytic polymer material, consisting of active particulate methane monooxygenases (pMMOs) embedded in polyethylene glycol diacrylate (PEGDA) hydrogel that converts methane to methanol under mild conditions with 100% selectivity. Firstly, they were able to embed PEG‐pMMO, via ultraviolet crosslinking, into a DIW printed 3D silicone lattice, with 250 µm struts and 250 µm void spaces. The resulting mechanically robust, gas‐permeable membranes were incorporated into a continuous flow‐through bioreactor, where methane/air mixture is introduced on one side, and the aqueous nicotinamide adenine dinucleotide (NADH, that supplies electrons to pMMO) on the other, with methanol continuously removed and collected in buffer (**Figure** **15**a). The methanol concentrations were 12% and 6% of those predicted for thin and thick membranes respectively, based on pMMO activity of 80 nmol MeOH min^−1^ mg^−1^. This work also involved directly 3D printing PEG‐pMMO material using projection microstereolithography (P*µ*SLA). A 3D printed PEG‐pMMO lattice structure with 100 µm^2^ vertical hollow channels in a 1000 µm^2^ hydrogel with a 500 µm height, as shown in Figure 15b, resulted in pMMO activity of 29 nmol MeOH min^−1^ mg^−1^. As a result of bending of struts in the lattice geometry due to water surface tension, the authors printed various solid and hollow PEG‐pMMO cylinders with surface area to volume ratios of 1.47–2.33 and diameters of 1–5 mm (Figure 15c, d). The highest surface area to volume ratio of 2.33 showed an average pMMO activity of 128 ± 14 nmol MeOH min^−1^ mg^−1^ per cylinder, which corresponds to the maximum activity of membrane‐bound pMMO with NADH as reducing agent, previously reported in literature.

Methanol derived using clean energy processes, as the one mentioned above, can be used as a fuel for fuel cells or can also be directly converted to value added products. For instance, Lefevere et al. employed DIW to fabricated various macroporous supports coated with thin layer of zeolite for methanol‐to‐olefin conversion.^184^ The design of DIW fabricated porous supports makes it possible to avoid a trade‐off between pressure drop and mass and heat transfer. When compared to conventional packed bed and honeycomb supports for conversion of methanol, AM fabricated supports showed excellent catalytic properties.

**Figure 16:**
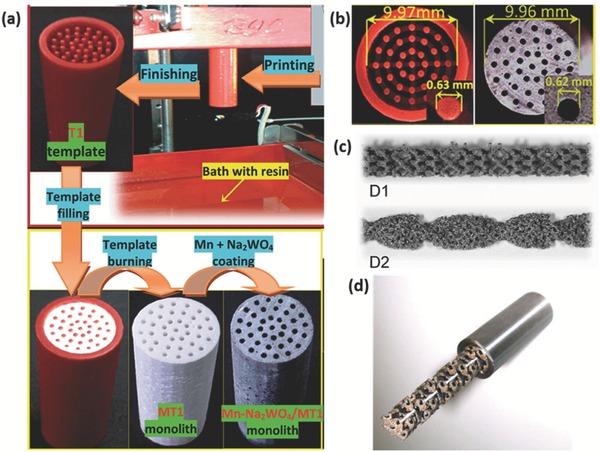
Schematics showing monolithic catalyst preparation. a) SLA printing of a polymer template (T1), followed by filling the template with α‐Al_2_O_3_ paste, burning of polymer template and coating the resulting monolith (MT1) with Mn‐Na_2_WO_4_, b) optical images of dimensions of SLA fabricated template and monolithic catalyst. Reproduced with permission.^186^ Copyright 2016, Royal Society of Chemistry. c) Catalytic static mixers (CSMs) fabricated via EBM. Reproduced with permission.^188^ Copyright 2016, Royal Society of Chemistry. d) Uncoated designed porous structure reactor (DPSR) fabricated via SLS. Reproduced with permission.^189^ Copyright 2015, Elsevier.

Methane derived from green sources, such as biomass,^185^ can also be directly converted to value added chemicals. Recently, Michorczyk et al. employed SLA to fabricate 3D polymer templates used in preparation of Mn‐ and Na_2_WO_4_ monolithic catalysts for the oxidative coupling of methane, as shown in **Figure** **16**a.^186^ The use of AM allowed the precise control of monolith channel architectures (Figure 16b) without variations in size during the burning of the template. The channel structure of this monolithic catalyst significantly influenced the catalytic performance of the oxidative coupling of methane, exhibiting 23–25% methane conversion and a high selectivity (67–70%) towards C_2_‐C_3_ hydrocarbons. Hydrogen produced from water electrolysis can also be used in various hydrogenation reactions.^187^ It was recently demonstrated that AM can be employed to directly fabricate novel 3D catalyst support structures for hydrogenation reactions, such as catalytic static mixers (CSMs),^188^ illustrated in Figure 16c, and uncoated designed porous structure reactor (DPSR),^188^ shown in Figure 16d. By using AM, limitations of classical sheet metal forming techniques can be overcome leading to a complete design freedom.^188^

#### 4.1.1 Challenges and Future Directions

In the aforementioned studies AM was mainly used to fabricate catalytic supports either directly or via templates. The subsequent coating or impregnation of catalyst layer introduces additional steps to the process of reactor fabrication. AM technologies, including DIW, inkjet printing and SLA are capable of direct fabrication of catalytic structures. However, there are certain obstacles that restrict this approach. For instance, a challenging issue in the use of SLA to produce fully functional catalytic reactors in a single step process, lies in the fact that mainly polymers can be processed by this technology. Whereas a wide range of active materials available for DIW and inkjet printing, is hindered by their relatively restricted geometrical freedom when compared to other AM technologies. However, the ability of DIW and SLS to control porosity is expected to deliver a big impact on their use in fabrication of chemical reactors. A number of reactions involved in chemical energy and fuel processing operate at high temperature and pressure, which makes metal based AM processes, such as SLS, very attractive for applications in such conditions.

### 4.2 Solar Cells and Photocatalytic Reactors

The absorber (i.e. semiconductor) thickness in solar cells is determined by a balance between light absorption, requiring larger absorber thickness, and charge collection, requiring smaller absorber thickness, with high purity absorbers allowing charge collection over the scales ranging from 10 nm to 50 µm, which is sufficient for efficient light absorption.^176^ Even though a number of AM technologies are capable of producing layers with thickness that fall into that range, the range of materials that fall into the absorber category is limited to DIW and solution based FDM methods. AM is more suited for fabrication of light trapping nanostructures (i.e. photonic crystals) and solar concentrators (micrometer‐sized parabolic mirrors, depicted in Figure 2c),^43^ which can be used to increase solar cell efficiency by minimizing entropy losses. In terms of macroscale design, AM shows potential for fabrication of optimized 3D solar panel frames,^41^ this circumvents the need for sun tracking, as was previously mentioned.

TiO_2_ nanoparticles are widely used semiconductor materials in photocatalysis. Ability to control the nanoscale structure of TiO_2_ is of vital importance to effectively harvest solar energy in photocatalytic applications. For instance, highly ordered TiO_2_ nanotube arrays with controllable length, demonstrate a considerable improvement in photogenerated electron transport, when compared to particulate TiO_2_ films, since this ordered architecture provides unidirectional electric channel and reduction in charge recombination.^190^ Hierarchical semiconductor structures with macro‐ and mesopores employed in photocatalysis, are another example of the importance of multiscale design. The presence of macropores results in light harvesting macrochannels that increase photoabsorption and enable efficient diffusion of gaseous molecules, the resulting light harvesting path allows photons to penetrate deep inside the surface of mesopores.^127^ Out of all AM technologies, DIW shows the most potential for the direct fabrication of semiconductor structures, however it would only offer the control of the macropore design. Optofluidics, a combination of microfluidics and optics, is another interesting avenue in photocatalytic applications, since microfluidic reactors can increase photocatalytic reaction rates due to improved mass and photon transfer efficiency.^147^, ^191^, ^192^ AM has proven to be very useful in microfluidics community in recent years, due to its ability to fabricate enclosed microchannels with complex geometries.^10^, ^193^, ^194^, ^195^, ^196^, ^197^, ^198^, ^199^ The precise control of microchannel size and geometry in photocatalytic reactors allows several advantages, such as large surface to volume ratios, ability to fine tune mass and light transport and uniform light distribution to the photocatalyst surface.^192^

#### 4.2.1 Solar Cells

**Figure 17:**
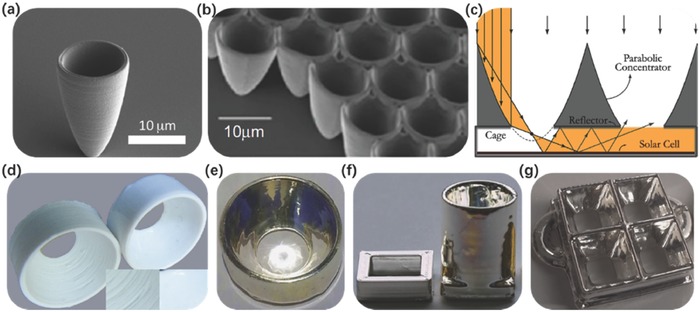
a) SEM image of single paraboloid coated with silver. Reproduced with permission.^44^ Copyright 2011, AIP Publishing LLC. b) SEM image of micro‐parabolic reflector array fabricated via 2PP in metal‐coated resist. Reproduced with permission.^43^ Copyright 2013, Nature Publishing Group. c) Schematic illustration of a parabolic concentrator array for external light trapping. Reproduced with permission.^200^ Copyright 2015, Elsevier. Optical images of a compound parabolic concentrator (CPC) fabricated via FDM — d) before and after chemical smoothing, e) after silver coating and (f) external light trap macrostrucutres. Reproduced with permission.^201^ g) FDM printed 2 × 2 square array of concentrators, used as an external light trap. Reproduced with permission.^200^ Copyright 2015, Elsevier.

Researchers at California Institute of Technology employed Nanoscribe's 2PP system to fabricate an array of microphotonic parabolic light directors made of photoresist (IP‐L).^44^ These microstructures, which are 22 µm high and 10 µm in diameter, as shown in **Figure** **17**a,b, the principle of which was shown in Figure 17c, were coated with 20 nm of chromium (Cr) and 380 nm of silver (Ag) using plasma sputter coating. Light transmission apertures with a 1.3 µm dimeter were etched into the bottom of parabolic light directors by employing a focused ion beam lithography. The experimental results indicated that these microphotonic (parabolic concentrator) structures are suitable for producing collimated beams for solar cell applications, improving solar cells efficiency by reducing entropy changes. The use of such micro light directors integrated to the surface of solar cells can result in ultrahigh conversion efficiencies, as the micromirrors can direct radiative emission from solar cells into a small solid angle, consequently reducing entropy loss.^43^, ^44^, ^45^

Light trapping is becoming increasingly important for nanocrystalline silicon (nc‐Si) solar cells, in order to efficiently couple light into PV cells and enhance light absorption. Unlike internal light trapping, external light traps leave material quality and electrical properties of the cell unaffected. The designs of such light traps can be rapidly and cheaply tested using AM. Dijk et al.^201^ recently demonstrated a 3D printed external light trap that is of interest for all solar cells, since it is placed as an add‐on on top of the cell and retro‐reflects the light that is reflected and radiated by the solar cell. The structural components of the light trap are printed via FDM and made of smoothened, silver‐coated thermoplastic (Figure 17d–f). A broadband absorption enhancement caused by applying one 3D printed external light trap on a 1 cm^2^ silicon (Si) solar cell and resulted in improvement of 15% of both photo‐current and power conversion efficiency in a thin‐film nanocrystalline silicon (nc‐Si:H) solar cell. FDM was can also be employed to print an array of concentrators, as shown in Figure 17g, in order to overcome issues associated with scaling up a single external trap to cover a large solar panel.^200^

Recently, Vak et al.^202^ reported on the use of modified FDM platform to fabricate solution‐processed organic solar cells layer by layer. The parts (bevel gears, brackets, a syringe holder and pusher block) used for modification were fabricated using FDM. The solution dispenser was mounted on an existing head and integrated syringe pump was designed to feed solution directly from syringe to nozzle without any tubes (to reduce cross‐contamination). Additional stainless steel mini slot‐die head was mounted on the printer, for making thin and uniform coatings. This so‐called “3D Coater” was used to fabricate fully printed bulk heterojunction (BHJ) solar cells (with a printed silver grid electrode) of various sizes onto ITO‐glass substrate. A module with 47.3 cm^2^ active area showed a power conversion efficiency (PCE) of 4.57%, which is the highest PCE for a solution processed OPV module reported in literature. The authors also demonstrated ability of their “3D Coater” to fabricate a fully printed perovskite solar cell, with PCE of over 11% and verified the possibility of low cost mass production of perovskite solar cells.^203^

#### 4.2.2 Photocatalysis and Solar Fuels

**Figure 18:**
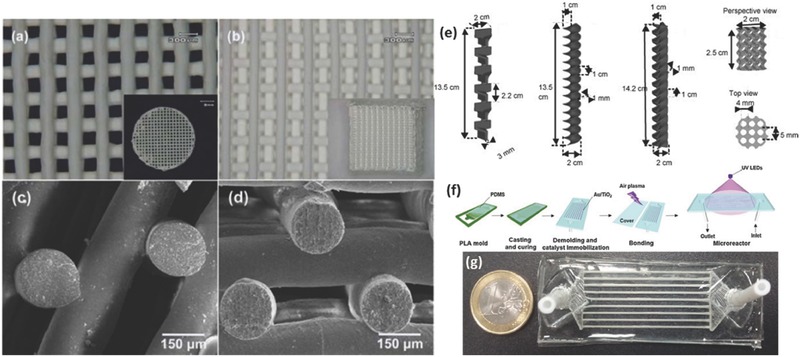
Optical images of DIW fabricated ZnO structures – a) cylindrical lattice with simple‐tetragonal symmetry with 10 layers and 200 µm rod spacing, b) square lattice with face‐cantered‐tetragonal symmetry with 20 layers and 566 µm rod spacing and SEM images of ZnO square lattice sintered at (c) 900 °C and (d) 1500 °C. Reproduced with permission.^208^ Copyright 2016, Elsevier. e) CAD images of the monolithic support designed and made by SLA: static mixer (SM), double spiral (DS), quadruple spiral (QS) and crossed channels (CC). Reproduced with permission.^207^ Copyright 2010, Elsevier. Silicone microreactor for photocatalytic generation of hydrogen – f) Schematics of silicone photoreactor fabrication steps. Reproduced with permission.^209^ Copyright 2016, Elsevier. g) Optical image of silicone microreactor loaded with Au/TiO_2_ photocatalyst. Reproduced with permission.^210^ Copyright 2017, Elsevier.

There are very few reports on AM manufactured photoelectrochemical flow cells. In fact, Achilli et al.^204^ were first to employ 3D printing for fabrication multi‐purpose photoelectrochemical devices for in situ and in operando experiments with synchrotron radiation. Two different designs of a photo‐spectroelectrochemical cell for investigating photoelectrochemical water splitting were manufactured using Polyjet 3D printer (Objet 30 Pro, Objet‐Strarasys), in a VeroWhite material (opaque rigid photopolymeric polyacrilic resin – stiff and chemically resistant). A layer resolution was set to 28 µm, which was appropriate for the electrolyte reservoir and reference electrode channels, with a diameter of 500 µm. The precision of 3D printing allowed controlling the thickness of electrolytic solution avoiding issues associated with X‐ray attenuation coefficient by water. Similarly, Bozhidar et al. used FDM to fabricate structural components of a gas phase photocatalysis reactor used for online monitoring of degradation of volatile organic compounds (VOCs), common air pollutants.^205^ Furman et al. also employed SLA to fabricated monolithic polymer support designs, shown in **Figure** **18**e, coated with TiO_2_ for photocatalytic degradation of VOCs.^206^, ^207^ These AM fabricated photocatalytic supports, which would not be possible to fabricate using classic manufacturing methods, allow photocatalytic reaction intensification by geometrical means via an increase in irradiated area.^207^

Recently, Castedo et al. employed AM in order to create a PLA mold used to fabricate a silicone microreactor for photocatalytic generation of hydrogen from bioethanol.^209^, ^210^ FDM printed mold consisting of nine rods of 500 µm (width) x 1 mm (depth) x 47 mm (length), with a total volume of 0.21 cm^3^ and two collectors to facilitate gas distribution, was cast with PDMS and cured, followed of by peeling off the silicone microreactor and coating with Au/TiO_2_ photocatalyst and finally sealing with thin silicone cover, as illustrated in Figure 18f.^209^ The resulting photoreactor (Figure 18g) was tested at room temperature and atmospheric pressure under various reaction conditions (photon irradiance, residence time, photocatalyst loading and water‐ethanol ratio). This approach could intensify photoconversion of bioethanol to hydrogen by allowing an efficient transport of photons, through a good contact between the photocatalyst and reactants and a good exposure to light.^210^

Another attractive strategy for production of solar fuels can be achieved by directly shaping semiconductor materials, such as TiO_2_ and zinc oxide (ZnO), which are commonly used for photocatalytic water splitting and CO_2_ reduction,^211^ into 3D structures. This can be enabled by certain AM technologies, particularly DIW. For instance, Tubio et al. prepared ZnO (48 vol%) inks to fabricate 3D periodic structures consisting of alternating tetragonal symmetries with well‐defined interconnected layers with rod diameters of 200 µm, as depicted in Figure 18a–d.^208^ DIW was also used to construct spiral, woodpile, lattice (Figure 9h), cylindrical and half‐conical TiO_2_ structures with micrometer scale dimensions.^160^, ^212^ Torres Arango et al. recently used DIW to deposit thin TiO_2_ films on flexible substrates, that were capable of photocatalytic degradation of methylene blue (MB), which highlights the potential of DIW fabricated TiO_2_ films in chemical degradation applications.^213^ It is however, 3D photocatalytic structures that show even more promise than thin films, as DIW fabricated α‐Fe_2_O_3_ woodpile structure with rod diameters of 250 µm was used for photocatalytic degradation of MB under UV light, and it was found that its photocatalytic properties are significantly improved compared to that of a bulk sample with same weight, due to a higher specific surface.^214^ Considering all of the above, DIW presents itself as an ideal AM technology for the fabrication of fully functional 3D photocatalytic devices.

#### 4.2.3 Challenges and Future Directions

AM showed very promising potential in solar cell applications, as was demonstrate by the direct and low cost mass production of organic solar cells via modified FDM/DIW coater; as well as fabrication of internal light traps (via 2PP) and external light traps (FDM). Moreover, AM can also be used to manufacture frames for 3D PV architectures, that circumvent the need for light trapping. In order to integrate all of the aforementioned developments within a single solar cell device, several obstacles need to be overcome. Some of these challenges were outlined in a recent review article by Ruiz‐Morales et al., which include the need for major advances in printable active materials to enable multi‐layer fabrication; developments in AM of flexible contact materials and high‐quality optics.^178^ Ruiz‐Morales et al. also suggested that AM has a future potential in fabrication of luminescent solar concentrators (LSCs), which are comprised of high quantum efficiency luminescent species within the highly transparent polymer matrix, used for re‐emitting absorbed incident light at a red‐shifted wavelength, which could become a cheaper alternative to silicon substrates in building integration applications of solar energy.^178^ SLA seems to be an ideal AM technology for LSCs production, as luminescent nanomaterials can be dispersed in methyl methacrylate resin. For this purpose, there is a need for SLA systems that would employ infrared laser, instead of UV or visible lasers, which are currently used to carry out photopolymerization, since luminescent species absorb UV and visible light and would compete with photoinitiators present in the resin. This would in turn require the development of novel photoinitiators that would initiate polymerization of methyl methacrylate upon exposure to the infrared light.

AM also proved to be very useful in photocatalytic applications, as was demonstrated by fabrication of novel architectures of photocatalytic supports, templates for photocatalytic microreactor fabrication and direct fabrication of porous semiconductor structures. Major advances in development of printable semiconductor inks and new strategies to further fine‐tune microstructure are still required. For the case of photocatalytic microchannel reactors, the recent advances in 3D printing of PDMS, can potentially be employed for direct fabrication of photocatalytic microreactors via SLA.^90^ However, further efforts in developing siloxane based resins are required, since in the previous study a dye was used to increase the resolution of printing, which in turn restricts the optical transparency of PDMS, which is essential for photocatalytic applications. Conductive polymers such as polypyrrole (PPy) and polyaniline (PANI) have shown a great deal of promise in photoelectrochemical splitting of water into hydrogen (H_2_) and carbon dioxide (CO_2_) reduction to fuels.^215^ Due to the low processability of conductive polymers, their applications and development are restricted. It is envisioned that development of 3D printable conducting polymer materials will advance the application of AM in solar fuel generation.

### 4.3 Fuel Cells and Electrolysis Cells

The control of electrode microstructure (composition, thickness, porosity and homogeneity) plays an important role in fuel cell applications. Hierarchically structured electrodes with electronically and ionically conducting phases and porosities at various length scales are capable of minimizing losses stemming from electrode kinetics and mass transport.^127^ Ability to control macroscale design of fuel cell flow field plates can result in enhanced mass transport properties and low catalyst loading, by ensuring uniform reactant distribution to the catalyst layer, whilst aiding water removal.^216^ In recent years, AM technologies found a widespread use in manufacturing of various electrochemical cell components, including helical stainless steel electrodes,^114^ titanium liquid/gas diffusion layers^217^ and polypropylene flow plates for proton exchange membrane (PEM) electrolyzer.^218^ These reports show a great deal of promise for the application of AM in fuel cell fabrication.

#### 4.3.1 Microbial Fuel Cells

**Figure 19:**
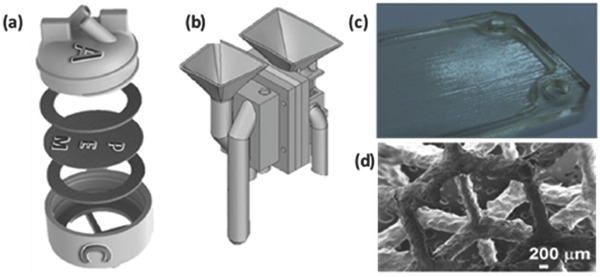
a) Twist n' Play' MFC design and (b) EcoBot‐III MFC design. Reproduced with permission.^222^ Copyright 2015, Elsevier. c) Tangoplus 3D printed membrane.^224^ d) Cellular AlSi10Mg anodic structure made by SLM as appears in Field Emission Scanning Electron Microscope.^225^

Due to low operating temperatures of Microbial fuel cells (MFCs), ranging from 4 °C to 35 °C,^219^ AM thermoplastics received a great deal of attention in fabrication of MFC structural components.^220^, ^221^, ^222^ Researchers at Bristol Robotics Laboratory (BRL) suggested that AM can facilitate the development of MFCs, by allowing a simple and rapid production of compact external structures with the absence of fixtures (screws and clamps), fittings and gaskets.^220^ Papaharalabos et al.^222^ demonstrated AM's capability of producing intricate reactor designs. In this work, a novel MFC‐chamber (‘*Twist n' Play*’) was 3D printed, as depicted in **Figure** **19**a, the variants of the same design were made from 3 different materials: medical‐grade biocompatible Polycarbonate (PC‐ISO) and ABS via FDM, ceramic‐filled photo curable resin (RC25) via stereolithography; and were compared with an established design made from RC25, as depicted in Figure 19b.^223^ The ‘*Twist n' Play*’ design outperformed the established model by a maximum of 74% in terms of power generation; from 3 different structural materials used, RC25 proved to be the most robust, whilst retaining better power and chemical oxygen demand (COD) treatment performance.^222^ Proton exchange membrane (PEM) is one of the key components in MFCs, since it facilitates ionic conduction, necessary for redox reactions to take place, and provides electrical separation between electrodes. Low cost and ease of fabrication are the driving forces in PEM material and manufacturing research, which led researches at BRL to manufacture Tangoplus polymer (its hygroscopic nature provides micro‐channels for ionic transport) ion exchange membrane (Figure 19c) via Polyjet 3D printing technology, with the aim of increasing specific proton exchange area for a specified anode chamber volume, whilst reducing overall dimensions of MFC by reducing the need for supporting structural material.^224^

Even though 3D printed PEM resulted in a significantly lower power output compared to the conventional latex cation exchange membrane (CEM), it was of the same order of magnitude and Tangoplus polymer showed absence of biodegrading and biofouling, which facilitated consistent maximum power and average energy output.^224^ It is believed that these benefits can contribute to the longevity and consistent performance of MFCs, however further research into ionically conductive properties of AM materials is required to reduce internal resistances which would result in a higher power output. The work carried out by BRL demonstrates that AM technologies can potentially be utilised to manufacture MFCs as a single unit.^220^, ^221^, ^222^, ^224^ This possibility was explored by Calignano et al., who used SLM to manufacture a bio‐inspired lattice aluminum alloy anode to improve the functionality of MFCs (which resulted in higher power densities in comparison to other metallic anodes) and FDM to construct the external structure, consisting of anode and cathode chambers, which could be opened with a non‐assembly mechanism.^225^ SLM fabricated anode (Figure 19d) possessed a 3D macroporous structure with ideal properties for hosting anodophiles, such as low density, large specific surface area and customized surface roughness. The cellular lattice structure of the anode resulted in a better microbial attachment and enhanced charge transfer between anodophiles and electrode surface, as well as good nutrient diffusion without clogging due to a network of open‐channels.^225^ The potential of 3D printing of carbon based cathodes^129^ can open up a possibility to fabricate fully AM‐based MFCs.

#### 4.3.2 Proton Exchange Membrane Fuel Cells

Generally PEMFC stack designs can be classified into three major categories: bipolar (from 100 W to 1 MW), pseudo bipolar (from 20 W to 150 W) and monopolar (low power output)^226^ In all of the aforementioned fuel cell stack configurations, flow field design plays an important role and can result in an increase in power density of as much as 50%,^227^ since flow patterns greatly influence reactants transport efficiency and therefore fuel cell concentration losses. AM shows a great deal of potential for PEMFC flow field fabrication, which was illustrated by Chen et al. who employed FDM to accelerate the fabrication of air‐breathing monopolar stack of miniature PEMFCs.^228^, ^229^ AM took significantly less time to manufacture the geometry of flow field plates (around 1 hr), when compared to traditional microfabrication processes, such as MEMs technology (12–36 hr) and CNC machining (more than 2 hr). Flow field plates were made of ABS, a material capable of sustaining corrosion of hydrogen proton and PEMFC operational temperature, as well as providing a high stiffness structure, which is essential to maintain low contact Ohmic resistance.^228^ The performance of this 10‐cell air‐breathing planar array PEMFC stack reached state of the art level, with peak power densities of 99 mW cm^−2^ at 0.425 V for parallel connected stack and 92 mW cm^−2^ at 4.25 V for serially connected stack under the conditions of ambient temperature, free convection air and 70 °C relative saturated humidity of hydrogen.

**Figure 20:**
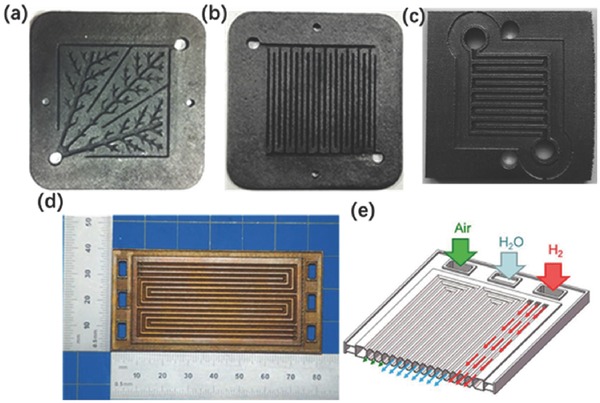
a,b) SLS fabricated graphite composite BPPs: a) interdigitated bio‐inspired design using Murray's law and (b) conventional interdigitated design. Reproduced with permission.^236^ Copyright 2014, Elsevier. c) SLS fabricated stainless steel anode plate. Reproduced with permission.^237^ Copyright 2015, Springer. d) DMLS fabricated titanium alloy BPP and (e) schematic representation of (d). Reproduced with permission.^238^ Copyright 2015, the authors. Published under CC‐BY‐NC‐ND 4.0 license.

In bipolar PEMFC stack design, bipolar plates (BPPs) are one of the key components and work as electron conducting materials separating membrane electrode assemblies (MEAs), providing flow channels for fuel and oxygen distribution and aiding water and heat management. As a result, BPPs can account for 40% of the total costs and 80% of the total weight of the stack.^230^ Early work on AM fabricated BPPs was carried out by Bourell et al.,^231^ who developed a novel indirect SLS process to fabricate graphite composite BPPs, in order to avoid expensive tooling costs of traditional BPP fabrication methods, such as injection molding (IM) and compression molding (CM). Laser sintered graphite and phenolic binder resulted in significant porosity. To densify the part and obtain gas impermeability, following post processing steps are required: 1) thermal phenolic dissociation cycle, which results in graphite particulates adhered by carbonaceous ligaments; 2) epoxy resin infiltration, which fills up most of the pores. Guo et al. utilized the aforementioned SLS based method to investigate the effect of different graphite materials on electrical conductivity and flexural strength of BPPs.^232^, ^233^ They found that carbon fiber addition to natural graphite and phenolic binder can lead to a 40% increase in flexural strength and a drop in electrical conductivity of BPPs, however for low carbon fiber additions, electrical conductivity satisfies US Department of Energy specifications, and that electrical conductivity and flexural strength values of BPPs fabricated via SLS are comparable to those fabricated using IM and CM. Furthermore, Guo et al.^234^, ^235^, ^236^ showed that multiple complex flow channels designs could be rapidly prototyped by using SLS. The novel flow designs inspired by the venation structure of a tree leaf and fabricated via SLS (**Figure** **20**a), showed 20–25% improvement in peak power density of PEMFC, compared to the conventional designs (Figure 20b), due to more uniform reactant distribution.^236^ The experimental results also showed that SLS manufactured BPPs can provide a fuel cell with a steady performance, by preventing PEMFCs from flooding.^235^

Although graphite is an excellent material for BPPs due to its chemical resistance and low density, there has been a shift from carbon BPPs to metal ones, as they are becoming a standard for PEMFCs and it is believed that metal BPPs can provide a path to low‐cost, compact fuel cell stack.^239^ Recently, Gould et al.^238^, ^239^ employed direct metal laser sintering (DMLS, a version of SLS) to prototype titanium‐alloy BPPs (Figure 20d, e) for aerospace applications, which were subsequently coated with a low‐cost, robust, conductive TiO_2_/gold coating to improve their corrosion resistance and minimize surface resistance. A single cell PEMFC stack comprised of 2 DMLS fabricated titanium BPPs performed similarly compared to the one with conventionally manufactured carbon BPPs, however a 40‐cell stack resulted in a 20% less than expected current density, which was caused by inadequate flatness of several BPPs.^238^ One of the largest contributions to cell resistance is the GDL to BPP interfacial contact resistance.^239^ SLM is another AM technology that can be used to fabricate metal BPPs, which was recently demonstrated by Dawson et al.^237^ In this work, experimental results showed that the contact resistance and PEMFC performance of SLM manufactured 316L stainless steel BPPs (Figure 20c) is similar to BBPs of the same design fabricated by conventional machining process (CNC milling machine), which implies that SLM can aid the development of different designs before committing to costly tooling.

#### 4.3.3 Solid Oxide Fuel Cells

In the last few years, AM has also been utilized to build solid oxide fuel cell (SOFC) microstructures. For instance, Hernandez‐Rodriguez et al.^240^ developed stereolithography based method to produce 3D microstructured ceramic materials for the fabrication of SOFC components. In this work, yttria‐stabilized zirconia (YSZ) powders were dispersed in a photosensitive polymer solution and polymerized under light irradiation layer by layer (with 20 µm layer thickness), followed by sintering of the 3D printed part at 1400 °C in order to sinter the ceramic particles together and evaporate the organic material. To test the applicability of this method for SOFC applications, they fabricated a thin YSZ layer that separates anode and cathode sides, with experimental results showing that electrical conductivity of this 3D printed layer is in a good a good agreement with typical results published for YSZ films (0.05 S cm^−1^ at 900 °C).^240^ Similarly, Shah's Group at Northwestern University devolved new ceramic based inks that can be used in a single 3D printer to create individual SOFC components, such as cathode, anode, electrolyte and interconnects.^241^ When a layer of such inks is printed, a highly volatile solvent in the mix instantly evaporates, whilst other solvents evaporate at a slower rate, leaving the layer hard enough to maintain its shape, but at the same time soft enough for the next layer to meld to it, after the printing process the finished part has to be fired at 1250 °C to make it denser and smoother. Researchers at the US Department of Energy's National Energy Technology Laboratory (NETL) are currently employing AM techniques to engineer more ideal cathode configurations in order to increase SOFC performance.^242^ This is achieved by using computer models and spray coating technique to automatically deposit solid and gas interfaces layer by layer, in order to optimize the location and intensity of power‐producing reactions. Brandon et al.^243^ identified SLS as a potentially promising technique to create highly defined porous microstructures in a SOFC anode. In this proof of concept study, Ni‐patterned electrode was laser sintered on YSZ substrate, however attempting to laser sinter more than one layer resulted in substrate cracking. The reader is referred to a review by Ruiz Morales et al.^178^ for more information on application of 3D printing in SOFCs.

#### 4.3.4 Challenges and Future Directions

AM was extensively used to explore novel designs of bipolar plates in PEMFCs. For SLS, manufacturing of intricate flowfield designs at sub‐mm scale is the main challenge, which could lead to failure in the current collector. As a result, the direct fabrication of metallic or graphite bipolar plate microchannels is restricted by the resolution of SLS. In case of MFCs, separate studies demonstrated ability of AM to fabricate all of the components required to assemble MFC: flow plates, 3D electrodes and proton exchange membrane. However, the possibility of a fully AM manufactured MFCs is hindered by the limited number of functional polymers that can be 3D printed. The application of AM in SOFCs fabrication is at a relatively nascent stage, with inkjet printing the preferred technology. Currently, the use of AM is restricted to fabrication of planar stacks, due to the geometric limitations of inkjet printing technology. This in turn, hinders the exploration of 3D configurations, that have a potential to increase specific power per unit mass and volume, at the cell and stack level.^178^ All of the above suggests, that further efforts are required in developing active polymer materials that can be processed by AM, as well as the need to increase the resolution of SLS in order to fully control the electrode porosity at microscale and to fabricate well‐defined microchannels. At the same time, efforts should be directed at the exploring the possibility of employing AM to fabricate microfluidic fuel cells, where the requirement for proton exchange membrane is eliminated by the co‐laminar flow of liquid fuel and oxidant streams.^147^ This can be potentially facilitated by employing SLA, which proved to be a reliable way of fabricating microfluidic devices, along with development of resins that can be used to produce 3D conducting polymer structures.

### 4.4 Batteries and Capacitors

Energy storing reactions, especially multielectron reactions that are difficult to achieve with micron sized active battery materials, often can become feasible and kinetically facile for the same material at nanoscale.^176^ Nanoscale designs can be implemented in order to integrate poor mixed‐conducting active material with its electron path, which can be achieved by employing networked CNTs or parallel 3D current collectors using nanofoams; and to minimize solid‐state transport lengths for ions and electrons.^176^ Batteries and electrochemical capacitors (supercapacitors), consist of two electrodes and one electrolyte. Their performance can be improved by utilizing hierarchically structured porous electrodes, consisting of well‐connected pores and walls with thicknesses at nanoscale, due to shorter diffusion pathways and increased surface area.^127^ In terms of overall electrode architecture, compared to planar electrodes, 3D structures provides much shorter diffusion length and smaller ionic transport resistance, and its macroporous structure can potentially double energy density and efficiently use limited available space.^244^ Ability of AM to fabricate 3D structures with microscale dimensions can be of great benefit in electrode fabrication. DIW presents itself as the most promising AM technique for battery and supercapacitor electrode fabrication, due to its ability to process highly porous carbon based nanomaterials into structured 3D architectures.^130^, ^244^

**Figure 21:**
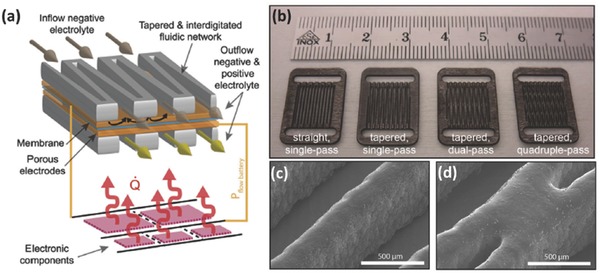
a) Illustration of a concept of a miniaturized RFB with an AM fabricated interdigitated fluidic network of tapered microchannels, which powers electronic components and the flowing electrolyte absorbs the generated heat; b) optical image of fluid networks with interdigitated microchannels fabricated via MJP followed by sputter coating 1 µm layer of nickel, c) SEM image of surface quality of AM fabricated structures, d) SEM image of a flow‐blocking element in the quadruple‐pass design. Reproduced with permission.^246^ Copyright 2017, Royal Society of Chemistry.

Similarly to fuel cells, AM has also been employed to fabricate flow field designs for hybrid zinc‐cerium redox flow battery (RFB).^245^ The FDM printed ABS electrolyte flow frames met the overall dimension tolerance of ± 10 µm, this precision is essential for the flow features in the inlet manifold of both frames and would have been hard to achieve by the traditional manufacturing methods. Recently, AM allowed Marshewski et al. to realize a novel energy conversion concept for a RFB, where power delivery and heat regulation are combined, thus RFB serves both as an energy conversion device providing electrical power to the electronics, and simultaneously as a heat exchanger for management of thermal load of electronics via liquid electrolytes, as illustrated in **Figure** **21**a.^246^ In order to increase power density of such RFB, mass transfer of electrolyte is optimized through the design and micro‐engineering of tailored fluidic networks, that follow an interdigitated layout of tapered microchannels that guide electrolyte in multiple passes through porous electrode whilst balancing pressure drops. These plastic interdigitated, tapered multi pass microfluidic networks (Figure 21b–d) with a total thickness of 600 µm, are fabricated via MultiJet printing (MJP) and subsequently sputter coated with 1 µm nickel (Ni) layer. Experimental results show that this novel RFB design concept can achieve maximum power densities of up to 14 W cm^−2^ at room temperature and net power densities (taking into account pumping power losses) of up to 0.99 W cm^−2^ for interdigitated microfluidic network in combination with tapered, multiple‐pass structures in alkaline electrolyte system. This represents a significant breakthrough compared to 0.45 W cm^−2^ (without taking into account the pumping losses) achieved using conventional serpentine design,^247^ which was previously the highest power density reported for alkaline RFB systems. In this novel RFB system, both active components: porous electrodes and ion exchange membrane were fabricated using conventional manufacturing methods. However, in addition to ability of fabricating ion exchange membranes mentioned in previous sections, AM has also found a use in production of functional components of static batteries, by utilizing the versatility of direct ink writing (DIW) technology, which allows for deposition of electrochemically active materials, which will be discussed further in this section.

#### 4.4.1 Zinc Batteries

In 2004, Malone et al. were first to report on AM fabricated primary zinc(Zn)‐air batteries.^248^, ^249^ The freeform fabrication system that employed syringe deposition for liquid chemicals and pastes, as well as fused deposition for plastics and metals, was developed for layer‐by‐layer manufacturing of a complete macroscopic battery, which consisted of copper(Cu)/silver(Ag) negative terminal, Zn/potassium hydroxide(KOH) anode, separator made of insulating ceramic foam, carbon black/manganese dioxide (MnO_2_)/KOH cathode catalyst, nickel(Ni)/Cu/Ag positive terminal and ABS casing.^248^ This freeform fabricated zinc‐air battery was able to power a small 30 mW DC brush motor for 2 seconds from only 1 g of zinc slurry. In order to address the issues of fragility and short operation time, the authors made changes to the formulation of zinc anode, cathode catalyst and separator materials which yielded consistent and stable rheology, allowing for reliable deposition, and improvement in electrochemical activity, which made it possible to produce Zn‐air batteries robust enough for quantitative characterisation.^249^ These improvements resulted in service lifetimes measured in days, as opposed to seconds, as well as in reliable power output of several milliwatts. However, these freeform‐fabricated batteries exhibited approximately 10% of power and energy densities of commercially produced Zn‐air batteries, which the authors attributed to electrolyte solvent loss, high internal resistances and poor catalyst activity.

In the following years, Ho et al. employed a custom made super inkjet printing (SIJP) system to fabricate 3D Zn‐Ag microbattery directly on a glass substrate.^250^ SIJP is capable of constructing sub‐micron dimensions with high precision, by ejecting volumes as small as 1 femtoliter, as a result of this this reduced drop volume, the solvent evaporates as the droplet travels towards substrate and is dried upon impact, which allowed the authors to construct two Ag electrode pads with arrays of 19 × 19 pillars. The printed silver pillars exhibited an average height of 40 µm and 10 µm diameters and were located 100 µm apart. The ink used in this work consisted of Ag nanopaste and n‐tetradecane. These 3D Ag pillars were subsequently sintered at 250 °C for 1 hr after printing. The Zn‐Ag microbattery was then self‐assembled in the charged state, by submerging a pair of Ag microstructures into an aqueous KOH electrolyte with dissolved zinc oxide powder causing surface of Ag positive electrode to oxidize, while Zn electroplates from Zn ions in the electrolyte onto the surface of the Ag negative electrode. The experimental results showed that the SIJP printed 3D Zn‐Ag microbattery configuration resulted in a capacity increase of 60%, compared to a cell with thin film electrodes of the same areal footprint, due to a volume increase of 50% and surface area increase of 20%.

In another work, Ho et al. developed zinc‐based chemistry compatible with DIW dispenser printer that can additively fabricate stacked, multilayer microbattery structures onto a substrate.^251^ The focus of this work was to develop solid‐state polymer electrolyte to eliminate the need to hermetically package any liquid components in the battery. Polymer solution composed of equal weight ratios of poly(vinylidenefluoride)‐co‐hexaflouropropylene (PVDF‐co‐HFP) and a 1‐butyl‐3‐methylimidazolium trifluromethanesulfonate (BMIM^+^Tf^−^) served as ionic liquid gel electrolyte. Whereas MnO_2_ and Zn were chosen as active materials for cathode and anode electrodes respectively. The rechargeable Zn‐MnO_2_ microbattery with an ionic liquid gel electrolyte was printed in a stacked configuration. The electrochemical experiments showed that the battery exhibited a discharge capacity of 0.98 mAh cm^−2^ and energy density of 1.2 mW h cm^−2^, as well as ability to be reversibly cycled more than 70 times without performance degradation.

#### 4.4.2 Lithium Ion Batteries

**Figure 22:**
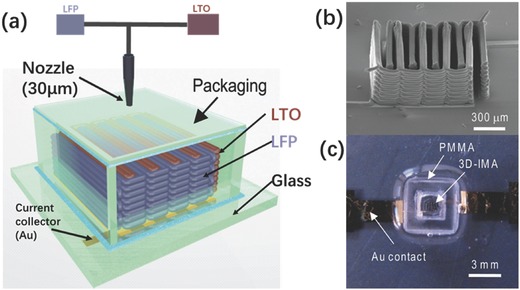
a) Schematic illustration of 3D interdigitated microbattery and the nozzle, b) SEM images of printed and annealed interdigitated LTO‐LFP electrode architectures, c) Optical image of 3D‐IMA composed of LTO‐LFP electrodes after packaging. Reproduced with permission.^254^

Considerable research efforts were undertaken on developing 3D electrode architectures of lithium ion (Lion) microbatteries in order to achieve high power and energy densities within a small areal footprint available to power microelectromechanical systems (MEMS).^252^ Currently, there is a lack of a simple and economic strategy to construct 3D Lion electrode configurations,^253^ however application of AM in 3D Lion battery microfabrication, shows a great deal of potential. For instance, Sun et al. used DIW to construct interdigitated secondary microbattery electrode architectures composed of lithium titanium oxide (Li_4_Ti_5_O_12_ or LTO) and lithium iron phosphate (LiFePO_4_ or LFP), which serve as anode and cathode materials respectively.^254^ Cathode and anode inks were prepared by suspending LTO and LFP nanoparticles in a solution of ethylene glycol, glycerol and cellulose‐based viscosifiers and deionized water via particle dispersion, centrifugation and homogenization. These inks were subsequently printed through a 30 µm nozzle (**Figure** **22**a) onto an interdigitated gold current collector that was previously patterned on a glass substrate. The printing process was followed by drying and annealing at 600 °C for 3 hr in an inert atmosphere and the resulting interdigitated LTO‐LFP electrode structure is presented in Figure 22b. Finally, the packaging of this Lion microbattery involved laser cutting PMMA preform, placing it around 3D printed LTO‐LFP electrodes, filling it with a standard liquid electrolyte and sealing with PDMS gel, as shown in Figure 22c.

To investigate the electrochemical performance of this electrode architecture, the discharge properties of printed 8‐layer and 16‐layer half‐cells composed of LFP and LTO electrodes were compared. The results showed a non‐monotonic variation in discharge capacity with electrode volume (number of layers), with the authors concluding that the functional height of the 3D printed electrode structure is limited by electron transport and kinetics of the reaction. A complete unpackaged 8‐layer printed Lion battery compares favorably against its rechargeable counterparts in terms of both areal power and energy density, demonstrating an areal energy density as high as 9.7 J cm^−2^ at a power density of 2.7 mW cm^−2^, due to high‐aspect structures that occupy a small areal footprint, while maintaining reasonably small transport length scales to facilitate facile ion and electron transport during charging and discharging. The capacity of a packaged LTO‐LFP microbattery was 1.2 mAh cm^−2^, however the packaged battery showed inhibited long‐term cycling due to the lack of hermeticity of the liquid electrolyte.

The evaporation and possible leakage of liquid electrolyte in microbatteries, can be eliminated by the use of solid‐state electrolyte, which was very recently demonstrated by Fu et al., who developed solid‐state electrolyte inks as well as GO‐based electrode composite inks to achieve fully 3D printed Lion microbatteries via DIW.^255^ The electrode inks were prepared by homogenously mixing chemically exfoliated GO sheets with LFP nanoparticles for cathode material and LTO for anode material and stored in separate syringes. The electrolyte ink consisted of a polymer composite containing a mixture of PVDF‐co‐HFP and aluminum oxide (Al_2_O_3_) nanoparticles. Due to the shear stress induced by the nozzle, the GO flakes are aligned along the extruding direction, which enhances the electrode's electrical conductivity; moreover, GO flake's intrinsically porous structure offers a large amount of surface to load the LFP or LTO nanoparticles as well as house the electrolyte. The printed interdigitated electrode structure is then freeze dried and thermally annealed to produce reduced GO (rGO), which is followed by printing of the solid‐state electrolyte into the channels between electrodes. The entirely 3D‐printed LTO/rGO–LFP/rGO microbattery can deliver initial charge and discharge capacities of 117 and 91 mAh g^−1^ and exhibit good cycling stability.

#### 4.4.3 Capacitors

Ho et al. proposed to employ a DIW technique for additively patterning of solid‐state, thick film carbon based supercapacitors directly onto a small autonomous electronic devices.^256^ They developed a gel electrolyte with high mechanical strength and good ionic conductivity consisting of 1‐butyl‐3‐methylimidazolium tetrafluoroborate (BMIM^+^BF_4_^−^) ionic liquid and PVDF polymer, and electrode made of mesocarbon microbeads (MCMB) active material and PVDF gel binder. The experimental results showed that these supercapacitors exhibited average capacitance of 0.5 mF cm^−2^. Subsequently, the same DIW platform was used to demonstrate the real life application of printed supercapacitors as ambient energy buffers to improve the lifetime of sensor nodes.^257^, ^258^ With the results indicating that DIW printed supercapacitors operate well using scavenged solar radiation and indoor light and show the potential of this platform to efficiently use the limited space available on the sensor board.^258^

**Figure 23:**
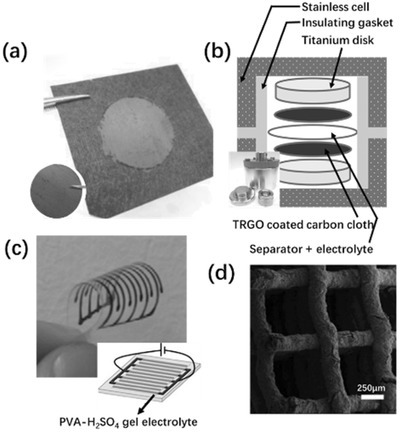
a) The printed TRGO electrodes on carbon cloth using 3D micro‐extrusion (insert: after punching for assembling); b) schematic illustration of the electrochemical capacitor assembly (insert: the photograph of the assembly). Reproduced with permission.^259^ c) planar micro‐supercapacitor printed on PET thin film (insert: illustration of the printed device). Reproduced with permission.^260^ Copyright 2015, Elsevier. d) SEM image of 3D hierarchical graphene aerogel electrode. Reproduced with permission.^261^ Copyright 2016, American Chemical Society.

Recently, Nathan‐Walleser et al. used a 3D micro‐extrusion technique to produce binder free thermally reduced graphene oxide (TRGO) based electrochemical capacitor electrodes.^259^ The pre‐printing steps consist of thermal reduction and exfoliation of GO, followed by high pressure homogenization (HPH) for production of printable, stable and highly concentrated TRGO inks in large quantities with the use of isopropanol solvent. Binder and surfactant addition was not required due to the presence of functional graphene sheets. TGRO inks were then printed onto electrically conductive carbon cloth via 3D micro‐extrusion, as depicted in **Figure** **23**a. The use of chemically robust carbon fabric as a substrate allowed the authors to test the performance of printed TRGO electrodes (16 mm diameter) in both aqueous 1 m KOH and non‐aqueous 1 m tetraethyl ammonium tetrafluoroborate‐acetonitrile (TEABF4‐ACN) electrolytes. The assembly scheme for TRGO based capacitors is presented in Figure 23b, in each device two TRGO electrodes are impregnated with electrolyte with a separator inserted in between and are packaged between titanium encapsulations. The assembled capacitors based on TRGO electrodes exhibited volumetric capacitances of 10 F cm^−3^ in 1 m KOH and 8.5 F cm^−3^ in 1 m TEABF4‐ACN. The TRGO based capacitor in non‐aqueous electrolyte showed high energy density of 4.43 mWh cm^−3^ and exhibited impressively power capability of up to 42.74 kW cm^−3^, these results are at least one order of magnitude superior to the values reported for high energy devices using graphene based materials.

Sun et al. also employed a self‐built 3D micro‐extrusion system to fabricate GO based planar micro‐supercapacitors layer‐by‐layer onto flexible substrates.^260^ Figure 23c presents the planar micro‐supercapacitor fabricated by DIW process, where GO ink is extruded to form an interdigitated electrodes pattern. In order to obtain all carbon solid‐state micro‐supercapacitors, printed GO electrodes are chemically reduced to rGO using hydroiodic acid (HI), which is followed by coating a layer of polyvinyl alcohol (PVA)‐sulfuric acid (H_2_SO_4_) gel electrolyte on top. In addition, this GO based planar micro‐supercapacitors method does not require the use of organic binder, conductive additives, metallic collector or polymer separator. To demonstrate the flexibility of these supercapacitors, they were printed onto thin PET films, the rGO films stably adhere to PET, due to large surface area and self‐alignment of graphene sheets. The printed micro‐supercapacitors showed high specific volumetric capacitance of 41.8 F cm^−3^, and exhibited high areal energy and power density of 7.6 mWh cm^−2^ and 29.2 mW cm^−2^ respectively, showing superior performance to micro‐supercapacitors fabricated by photolithography and laser patterning processes.

Even though it clear from the research on microbatteries that configuring the micro energy device components in 3D architectures can significantly improve the electrochemical performance of a device, there were relatively few studies on micro‐capacitors with 3D features.^262^ However, AM showed some potential in this regard. For instance, Zhao et al. used SLM to manufacture titanium alloy (Ti_6_Al_4_V) interdigitated micro‐supercapacitor electrodes.^263^ The top electrode consists of 32 vertical posts and the bottom electrode has 33 posts, with each post having a 500 µm diameter and an 8 mm height. Both SLM manufactured electrodes were coated with a polypyrrole (PPy) thin film via electro‐polymerization, and then filled with PVA–phosphoric acid (H_3_PO_4_) polymer electrolyte. This 3D printed electrodes configuration showed a comparable volumetric capacitance (2.4 F cm^−3^) to the 3D electrodes fabricated via lithography. The SLM printed 3D micro‐supercapacitor can provide a maximum energy and power density of 213.5 Wh m^−3^ and 4 kW m^−3^ respectively, in the current density range of 3.74–37.4 mA cm^−3^.

Very recently, Zhu et al. employed DIW to fabricate 3D periodic graphene composite aerogel supercapacitor electrodes (Figure 23d).^261^ They developed printable ink by adding graphene nanoplatelets (GNP) to GO in order to improve conductivity without detrimental lose in surface area. Hydrophilic fumed silica was also added to the GO‐GNO composite to serve as viscosifier to meet rheology requirements, and resorcinol‐formaldehyde (R‐F) solution was added to induce gelation post‐printing. The post‐print treatment steps involve gelation, supercritical drying and carbonization in order to process 3D printed structure into aerogels, followed by etching of silica with hydrofluoric acid (HF). The quasi‐solid‐state symmetric supercapacitor assembled with GO‐GNP‐SiO2 electrodes (1 mm thick) exhibited capacitance retention (90% retention when current density is increased from 0.5 to 10 A g^−1^), volumetric energy density (0.14 mWh cm^−3^) and volumetric power density (2.643 kW cm^−3^) equal to or exceeding those pf reported devices made with electrodes 10–100 time thinner. The authors attributed these electrochemical improvements to the development of GO and GNP composite inks and partly to the benefit of using 3D printing to create periodic macroporosity to facilitate mass transport.

#### 4.4.4 Challenges and Future Directions

Battery fabrication is by far the most promising energy application of AM. Firstly, the number of publications on the use of AM to fabricate battery components is significantly larger compared to other applications, with the first report on this matter dating back to 2004. Secondly, it fully utilizes the geometrical advantage offered by AM, which allows fabrication of high aspect ratio 3D porous electrode configurations. Thirdly, AM is capable of fabricating all the essential components required for a microbattery, including solid‐state electrolytes, which implies that in the near future, it can meet the demand for light weight, safe, high capacity and compact energy storage devices used in portable electronic devices. In a review article, titled “*Progress in 3D Printing of Carbon Materials for Energy‐related Applications*”, by Fu et al., the authors envision that AM can help to improve the energy storage performance and transition from conventional battery manufacturing methods to low cost and simple method, offered by DIW.^130^ Similar benefits offered by AM, apply to fabrication of supercapacitors. However, in order to achieve these goals, further efforts on development of advanced energy storage materials with improved mechanical properties, small particle size and high viscosity are required, which could result in high rate capability and long cycle life.^244^

### 4.5 Carbon Capture

Adsorption of CO_2_ by solid sorbents (zeolites, metal organic frameworks and nanoporous polymers) presents itself as a lower cost alternative to CO_2_ capture using amine solutions. Precise control over the sorbent materials design at nanoscale can enable atomic or molecular scale engineering of CO_2_ binding properties, such as enthalpy of adsorption, as well as critical transport properties, such as diffusion coefficients that would directly determine the efficacy of adsorbents.^176^ These materials can be structured into hierarchically porous monoliths, which demonstrate characteristics over the single‐mode porous components, as a result of macroporous network enabling improved mass transport properties and mechanical stability, whilst mesopores enable functionality for CO_2_ adsorption.^264^ This hierarchical approach presents a great opportunity for development of scalable solid‐gas contactors for cost‐effective CO_2_ capture. As was previously discussed, DIW is more than capable of fabricating, such monoliths. Microencapsulation of liquid sorbents within highly permeable shells, combines advantages offered by liquid sorbents, such as high capacity, high selectivity and tolerance to water, with advantages offered by solid sorbents, such as high surface area.^265^ These microcapsules are produced using flow‐focusing microfluidic devices, which can be easily fabricated by SLA and 2PP, due to the ability of these 3D printing technologies to fabricate enclosed microchannels with intricate geometries.

**Figure 24:**
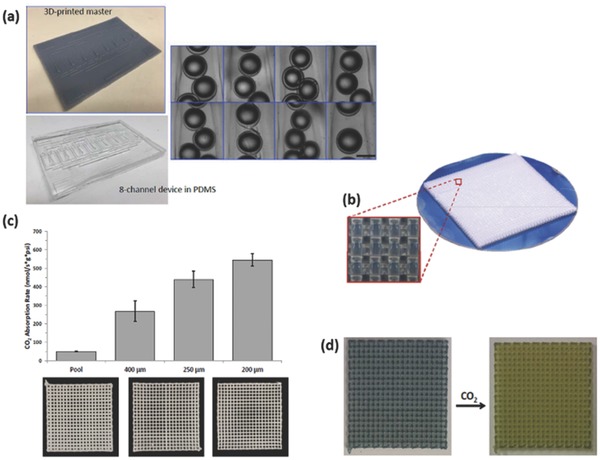
a) 3D printed master and finished 8‐channel PDMS hydrodynamic flow focusing device with the maximum throughput of 1 L/day. Reproduced with permission by NETL, 2016.^267^, ^270^ b) DIW printed tubes filled with carbonate solvent. Reproduced with permission by NETL, 2015.^268^ c) DIW printed sorbent‐polymer lattices with varying strut thickness.^267^ d) DIW printed sorbent‐polymer lattice with color indicating dye to identify CO_2_ loading. Reproduced with permission by NETL, 2016.^267^

In the last few years, AM gained some attention in CO_2_ capture applications. Bara et al.^266^ hypothesized that 3D printing can be utilized in fabrication of components and devices for CO_2_ absorption with liquid solvents. They demonstrated 3D printing of prototypes of packing materials, trays and entire gas‐liquid contactors. However, the experimental work on their performance, in terms of determination of mass transfer rates for CO_2_ absorption with aqueous sodium hydroxide is pending. Researchers at Lawrence Livermore National Laboratory, carried out extensive work on micro‐encapsulation of CO_2_ sorbents, as well as printed sorbent‐polymer composites, in order to increase surface area for CO_2_ capture.^265^, ^267^, ^268^, ^269^, ^270^ The authors realized the potential of advanced manufacturing techniques, that can enable development of new solvents and sorbents for more energy‐efficient and capital efficient carbon capture systems.^268^ For instance, they employed 3D printing for fabricating masters to rapidly prototype parallelized 3D hydrodynamic flow focusing microfluidic devices used to scale‐up the production of Micro‐Encapsulated Carbon Sorbents (MECS), as shown in **Figure** **24**a.^267^, ^270^ Another way to capture CO_2_ is via DIW of carbonate particles embedded within a CO_2_ permeable polymer (mainly silicones), as depicted in Figure 24b–d.^267^, ^268^ The lattice geometries can be optimised for gas flow and reactor shape, with smaller struts yield higher absorption rates.^267^

**Figure 25:**
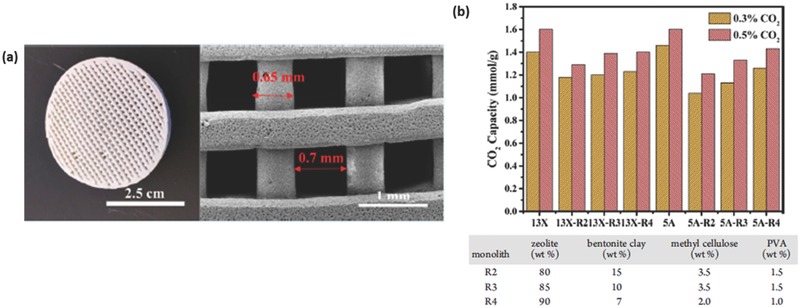
a) 3D printed zeolite monolith (left) and its SEM image (right); b) comparison of CO_2_ adsorption capacities between 3D printed zeolite monoliths and zeolite powders obtained at 25 °C using 0.3% and 0.5% CO_2_ in N_2._ Reproduced with permission.^271^ Copyright 2017, American Chemical Society.

Recently, Thakkar et al.^271^ reported the first example of using 3D printing to fabricate zeolite (13X and 5A) monoliths, as shown in **Figure** **25**a, for CO_2_ adsorption from air. These structures, with high zeolite loading (90 wt%), possess a network of microspores, mesopores and macropores of zeolite and binders, and show CO_2_ adsorption and adsorption capacity comparable to their powder forms (Figure 25b). The authors suggest that 3D printing allows and alternative approach to adsorbent material fabrication, with a capability to fine‐tune structural, chemical and mechanical properties for use in gas separation processes, and completely avoid the issue of attrition that is common with powder adsorbents. Inspired by these results, they were also able to 3D print aminosilica adsorbents into 3D monoliths, which exhibited CO_2_ adsorptive characteristics comparable to their corresponding powders.^272^

#### 4.5.1 Challenges and Future Directions

Similar to the application of AM in heterogeneous catalysis, DIW is capable of direct fabrication of 3D adsorptive monoliths. So far, there is a limited number of materials reported to have been processed via AM for CO_2_ capture. Therefore, further research into developing printable sorbent materials, such as zeolites, metal organic frameworks and nanoporous polymers, is required. At the same time, novel additive nanomanufacturing technologies, that can process sorbent materials, need to be developed, in order to gain a full control of design of mesopores. In case of production of MECS, via parallelized 3D hydrodynamic flow focusing microfluidic devices, new siloxane resin formulations that can be used in SLA need to be developed, in order to directly fabricate PDMS microfluidic devices and circumvent the need for fabrication of templates.

### 4.6 Thermal Energy Conversion

In thermal energy applications, nanoscale design can be used to develop materials with controllable thermal transport properties, for instance, graphene and CNTs have intrinsic thermal conductivities along the plane, comparable to that of diamond, however large contact resistance of such nanostructures need to be resolved.^176^ Precise control at larger scales is required to ensure large surface area available for heat transfer. Metal AM technologies, such as SLS, are good candidates for thermal energy conversion applications, due to their ability to fabricate complex 3D geometries, that can significantly increase surface available for heat transfer.

Norfolk et al.^273^ used a novel AM approach – ultrasonic additive manufacturing (UAM), in which ultrasonic welding is employed to bond a succession of metal tapes and subsequently fabricate complex 3D structures. Several structures were fabricated by this method with microchannels made of copper and aluminum, tested to find their burst pressures, which were demonstrated to be in excess of 27.60 and 20.68 MPa (4000, 3000 psi) respectively. Thus the operating pressure could be set at 6.90 Mpa (1000 psi). The conductivity of the two parts proved to be similar to the wrought ones. Fasano et al.^274^ employed SLS to fabricate the Pitot tube incorporated with heat sink, which lead to a 98% improvement in heat transfer compared to the conventional heat sinks. In this experiment, the AlSiMg powders were used and optimized parameters were set to obtain a low bulk porosity value (<0.8%), for the manufactured Pitot tubes. The heat sinks were tested in the fully developed turbulent regime of an open loop wind tunnel, with the hydraulic diameter of 187 mm, the average air velocity ranging from 3 to 15.5 m s^−1^, and the kinematic viscosity of 1.6 × 10^−5^ m^2^ s^−1^. The results showed that the 3D printed heat sink, at the open condition, exhibited the highest thermal transmittance than the closed one (32% in average). This was due to a secondary airflow and the interference of a boundary layer. While the reference heat sink presented the poorest heat transfer performance (50% ca of the 3D printed one in the open configuration), due to the absence of the secondary flow as well as the additional heat transfer area.

**Figure 26:**
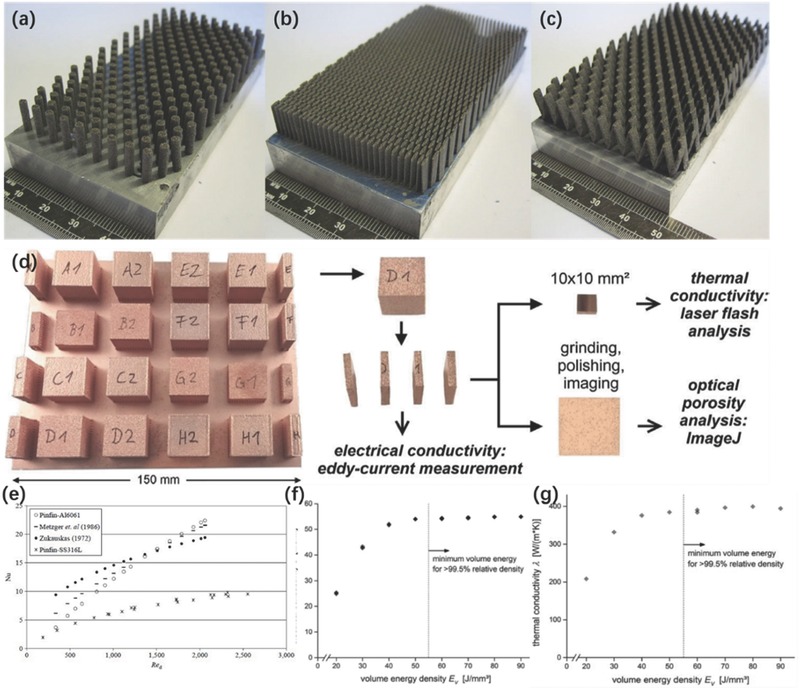
AM fabricated heat sinks (a) Pinfin‐Al6061; b) Diamond‐Al6061; c) V‐Al6061. Reproduced with permission.^275^ Copyright 2007, Emerald Group Publishing ltd. d) Procedures for SEBM fabricated copper sample preparation. Reproduced with permission.^277^ e) Heat transfer performance of Pinfin, Diamond and V geometries investigated. Reproduced with permission.^275^ Copyright 2007, Emerald Group Publishing ltd. f) Electrical and (g) thermal conductivities of SEBM fabricated copper samples with respect to volume energy density (various relative densities). Reproduced with permission.^277^

Wong et al.^275^ used 316L stainless steel and aluminum 6061 (particle size ranged from 10 to 45 µm) as raw materials to fabricate heat sinks using selective laser melting (SLM), SLS based AM method, with the focused beam size of 45 µm in diameter at 80 W power in Argon atmosphere and the layer thickness of 50 µm. 3 different heat sinks designs were fabricated out of aluminum via SLM: Pinfin‐Al6061 that has a conventional geometry (**Figure** **26**a), V‐Al6061 is constructed from angled ellipses (Figure 26b) and Diamond‐Al6061 from diamond shape pins (Figure 26c). Both Diamond‐Al6061 and V‐Al6061 designs possess geometrical properties that prevent their fabrication using conventional manufacturing methods. The structures were compared in heating airflow, and their heat transfer performance is presented in Figure 26e. The Diamond‐Al6061 design exhibited the largest improvement in heat exchange. Consequently, complex fins could enhance the thermal transfer by increasing the surface area. The comparison between Pinfin‐SS316L and Pinfin‐Al6061 illustrated that Aluminum 6061 was a viable material for heat exchange by SLM technology. In another work, Wong et al.^276^ demonstrated further SLM's ability to fabricate complex 3D heat sink designs, with features that are difficult if not impossible to construct using conventional manufacturing. The experimental results showed that increase in heat transfer area alone does not necessarily improve heat transfer performance, with coolant path through the heat sink geometry also playing a significant role.

SEBM (selective electron beam melting) method was used by Guschlbauer et al. to manufacture copper parts aimed to investigate their thermal and electrical conductivity performance.^277^ Raw materials, spherical copper powders with a particle size of 40–105 µm, were sintered at various volume energy densities (from 20 to 90 J mm^−3^) yielding relative densities ranging from 82.45 to 99.95%. Then the printed parts were cut and ground to test the electrical and thermal properties by eddy‐current measurement and laser flash analysis device, as illustrated in Figure 26d. The results shown that the electrical conductivity up to 55.82 MS m^−1^ (relative conductivity of 96.24%) and thermal conductivity of 400.1 W m^−1^ K^−1^ (relative conductivity of 99.78%) were achieved (Figure 26f, g).

**Figure 27:**
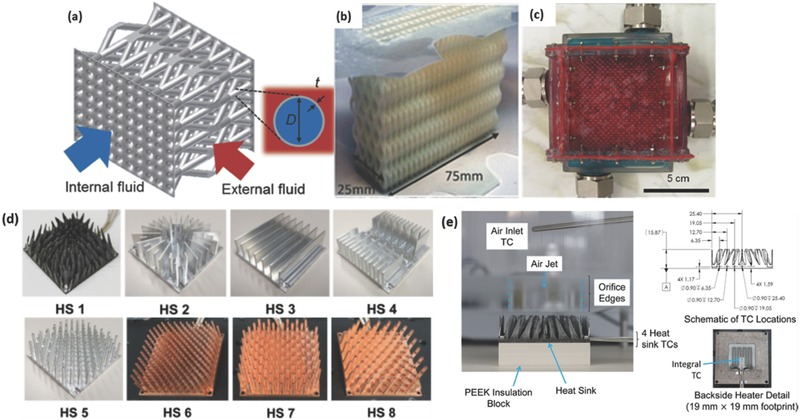
3D bicontinuous fluid networks for heat exchange — a) schematic representation of a bicontinuous fluid network, b) optical image of a single hollow lattice with sacrificial scaffold formed via AM used for fabrication of bicontinuous fluid network for heat and mass exchange and (c) optical image of bicontinuous fluid network with transparent headers and casing, with the blue dye flowing in the internal fluid domain and red dye in the external fluid domain, demonstrating interpenetrating, leak‐free domain. Reproduced with permission.^278^ d) Heat sink designs: HS 1 fabricated out of AlSi12 via AM, HS 2–5 out of 7075 aluminum via conventional machining and HS 6–8 out of oxygen free copper via conventional machining and (e) optical image of AM fabricated heat sink with 38.1 mm het orifice diameter (left), schematic illustration of thermocouple (TC) hole locations (top right) and installed heat sink backside heater detail (bottom right). Reproduced with permission.^47^ Copyright 2015, American Society of Mechanical Engineers ASME.

Recently, Roper et al. presented a scalable approach to yield nature inspired 3D bicontinuous fluid networks with thin‐walled interfaces beyond the limits of current materials approaches, enabling polymer heat exchanger designs (**Figure** **27**a,c) in which thermal performance is independent of thermal conductivity of the structural material.^278^ These complex 3D heat exchanger designs were realized by fabrication of sacrificial molds via SLA, as shown in Figure 27b, followed by conformal coating with parylene AF‐4 and selective removal of sacrificial scaffold. The design flexibility of AM alongside thin, conformal coating facilitated the combination of transport enhancing features, such as very large hydraulic diameter to wall thickness ratios (D/t), ranging from 5 to 30,100, and small D (13 to 0.09 mm), which cannot be achieved by conventional planar fabrication techniques, such as soft lithography and micromilling. Optimal design of D/t ratios in AM enabled efficient bicontinuous heat exchanger with prime surfaces allows the use of materials with low thermal conductivity, which can be selected for other material properties, such as electrical conductivity, antifouling ability or density. This in turn, allows the interface material to be selected for multifunctional applications, besides heat transport.

As previously mentioned in the Introduction, Dede et al. employed SLM to fabricate out of AlSi12 a novel heat sink design (HS 1), shown in Figure 27d, e, with sophisticated external features that are very challenging to fabricate using traditional manufacturing techniques.^47^ Its perfomance in terms of heat transfer and fluid flow was subsequently tested in confined jet impingnation cooling, and was compared to benchmark plate and pin‐fin heat sink designs (HS 2–8), illustrated in Figure 27d. The experimental results show that the AM fabricated novel heat sink design has higher COP relative to benchmark designs. This improved performance is attributed to the unique blended fin design leading to a reduced jet flow resistance.

#### 4.6.1 Challenges and Future Directions

The biggest challenge faced by utilization of AM in thermal energy applications, lies in the fact that metal AM technologies fabricate structures with internal porosity, which can have a detrimental effect on the thermal conductivity of heat exchanger. This would require further work to be carried out on the strategies to minimize porosity, either by developing new materials, introducing a number of post‐processing steps or technical improvements in metal AM technologies. Alternatively, AM shows a great deal of potential in fabrication of nature inspired 3D bicontinuous fluid networks with thin‐walled interfaces beyond the limits of current materials approaches, enabling polymer heat exchanger designs in which thermal performance is independent of thermal conductivity of the structural material, thus making it possible to utilize SLA and 2PP in fabrication of polymeric heat exchangers with fine features.

## 5 Perspectives

From previous sections, it is evident that AM has an enormous potential in rapid prototyping of novel and complex designs and facilitating research and development (R&D) of energy conversion and storage devices. In the last decade, patents for a number of key AM technologies, mainly FDM and SLA, had expired, which led to a significant number of affordable desktop 3D printers being released into to the market. This development in turn allowed more research facilitated by AM, which is evident from an increasingly growing number of publications that report on utilizing AM in in microfluidics as well as energy field. However, there are certain barriers that prevent it from being a truly disruptive manufacturing technology capable of fabrication of end‐products for commercial use, particularly in the energy field. This section is intended to briefly overview recent advances that have a potential to overcome these barriers and facilitate the use of AM fabrication of multi‐functional energy devices.

### 5.1 Material Development

A limited number of commercially available materials can be processed via AM, which are mainly restricted to polymers and certain metals and alloys. As previously mentioned, commercially available FDM and SLA polymers suffer from low temperature resistance and the absence of electrical conductivity, which restricts their application in energy devices. This drawback was realized by several 3D printing companies, for instance 3D Graphene Lab inc. introduced graphene conductive filament for FDM,^279^ with volume resistivity of 0.6 Ω cm, which is the most highly conductive filament on the market. Very recently, Formlabs developed a High Temperature Resin for SLA,^280^ which fabricates acrylic based polymer with the heat deflection temperature (HDT) of 289 °C at 0.45 MPa, which is reported to be the highest HDT of any polymer available on 3D printing market. Despite the obvious savings from less material waste, the cost of feedstock AM materials is another significant roadblock. For example, the acrylic based resin for a SLA printer is significantly more costly than a sheet of PMMA that can be machined. However, the price of materials seems to have started to decrease recently, with different start‐ups introducing their materials into the 3D printing market at a lower price than the established 3D printing companies do. More importantly, development of active energy materials is a nascent area of research in AM. However, in recent years there has been an increased activity of the research community in functional AM materials development, ranging from carbon‐based and metal‐based nanoparticles to zeolites and ionic liquids, for energy conversion, storage, transport and CO_2_ capture. The combination of the decreasing costs of structural AM materials and the increasing research interest in energy materials development is envisioned to facilitate the application of AM in fabrication of fully functional energy devices and bringing it closer to the commercial stage.

### 5.2 Multi‐Material, Multi‐Process AM

In In this review, we have predominantly addressed the use of multiple materials in AM, where either 1) the starting materials are pre‐mixed or composited prior to the AM process, or 2) the second material is integrated by infiltration, impregnation, coating or other non‐AM post‐processing techniques. The use of DIW with multiple nozzles to fabricate different layers of electrochemical and photovoltaic devices was also discussed. *Multi‐material AM* is a term classified by Vaezi et al.^281^ as a process in which “*different materials or chemicals are physically delivered to any spatial location in 3D during additive manufacturing” and* where compositional variation can be freely controlled by computer and a program. Ability for multi‐material fabrication is essential for AM utilization in energy applications, for instance photovoltaic cells and solid oxide fuel cells require multiple layers of active materials. Currently, DIW and inkjet 3DP are the only AM techniques able to meet this requirement. However, it was demonstrated that several AM methods can be combined within the same machine that is capable of fabricating multi‐functional 3D structures in a sequential layer by layer process that combine mechanical, chemical, thermal, electronic and other functions in a single component.^281^ Another strategy, known as *Multi‐process 3D printing*, to enable AM to fabricate multifunctional structures with multiple materials, involves introduction of robotic placement of components and complementary techniques, such as micromachining, dispensing of functional inks and embedding of wires into AM process.^282^ This combination of AM with complementary processes can provide spatial control of multiple materials, geometry and functionality. In turn, by merging the multiple components into a single structure, the time‐consuming assembly steps can be eliminated from the manufacturing process.

### 5.3 Additive Nanomanufacturing

**Figure 28:**
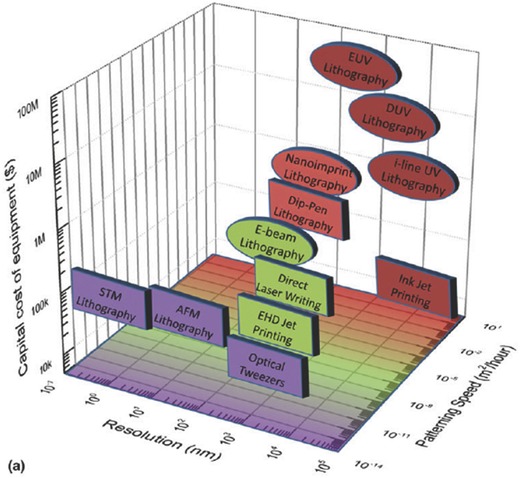
Additive nanomanufacturing methods (square) compared with conventional resist‐based lithography methods (ellipse) in terms of equipment cost, patterning speed, and minimum resolution. Reproduced with permission.^150^ Copyright 2014, Cambridge University Press.

The resolution of commercially available AM technologies needs to be significantly improved, in order to enter the realm of nanofabrication, to fully harness advantages offered by nanoscale design. Nanofabrication is classified as a set of processes and methods capable of producing nanostructures and devices with minimum dimensions lower than 100 nm.^283^ 2PP is currently the only commercially established AM technique with capability of fabricating structures with resolution of 40 nm and is mainly restricted to photo‐curable polymers. However, most energy applications covered in this review, require material design at sub nanometre scale (0.1‐several nm). Energy storage takes place at nanoscale, predominantly in energy bonding one atom to another, and cannot be separated from energy conversion and transport, therefore matching the time scales of energy carriers by controlling their characteristic length scales and propagation velocities is essential in nanofabrication design for energy conversion and storage.^176^ Rational design of catalytic centers requires precise control of structural features (active site and support structure) of energy materials. For instance, some traditional nanofabrication methods are capable of constructing CNT channels with inner diameters ranging from 0.8–8 nm that can accommodate catalyst nanoparticles (<1–5 nm), which allows the modulation of properties and behavior confined molecules and nanomaterials.^284^ Nanoimprint lithography (NIL), one of traditional nanofabrication methods, is tipped to become a practical method for large scale manufacturing of energy devices.^283^ However, the cost of traditional subtractive nanofabrication equipment is prohibitively expensive, with NIL in particular requiring capital costs that exceed $5 million.^150^ Additive nanomanufacturing (ANM) is a relatively nascent area of research that is aimed to bring AM closer towards nanofabrication. ANM technologies, such as dip‐pen nanolithography (DPN) and electrodynamic (EHD) jet printing, are able to achieve sub 30 nm resolution and have a capacity for 3D nanofabrication of a wide selection of materials, ranging from metals to organic materials.^150^ Despite the fact that the cost of ANM machines are significantly higher than the commercially established AM technologies, their estimated capital equipment costs are generally much lower compared to traditional subtractive nanofabrication methods, as shown in **Figure** **28**. This coupled with ability to fabricate structures with sub 30 nm dimensions, suggests that the development of AM technologies can enable AM to compete with traditional nanofabrication techniques sooner rather than later.

### 5.4 Novel Design Concepts and Strategies

In the past, design and optimization of energy devices was limited to the 2D domain due to the constraints of conventional manufacturing methods. With AM offering an almost complete design freedom, there is a need for development of new 3D design concepts. Examples of where novel design concepts were tested by employing AM, include validation of various optimized membrane module designs^17^ and fabrication of bio‐inspired structures with multifunctional features,^13^ which were previously inaccessible by traditional manufacturing. This is all possible due to the intrinsic ability of AM to control local microstructure and chemical composition. For energy field to fully harness the advantages offered by AM, novel design strategies need to be developed. Small steps in this direction were taken by the scientific community in the last few years. For instance, the use of topology optimization, an approach where geometry topology is not defined prior to optimization, but instead is allowed to develop as part of an additive design process involving a number of sequential simulations, for the design of complex 3D heat sink structures which can be fabricated using AM was recently demonstrated.^47^, ^285^ Design concepts can be produced quickly by the topology optimization approach in comparison to traditional approaches using parametric analysis.^47^ It can also be applied to other energy processes, since optimizer can generate geometry based on various objective functions, such as maximizing power and energy densities, minimizing electrical resistance, maximizing surface area for photon transport and mass transport.

Another design strategy can take form of integration of multiple energy processes within one system. As was previously mentioned, Marshewski et al. to realized a novel energy conversion concept, where RFB serves both as an energy conversion device providing electrical power to the electronics, and simultaneously as a heat exchanger for management of thermal load of electronics via liquid electrolytes, by combing power delivery and heat regulation.^246^ Moreover, Marshewski et al. suggest that this concept of combining functions in RFBs is not limited to just power delivery, they envision that the such RFBs can be integrated with energy harvesting devices, such as photovoltaic cells, whose performance decreases with increasing temperatures, and as a result could benefit from the cooling capacity of an RFB cell.^246^

Challenges in terms of the need for new design strategies due to seemingly unrestricted geometry freedom offered by AM, which impose different types of constraints and require different process specific tools and rules that are significantly different from conventional manufacturing processes, were also recently discussed in several review articles.^286^, ^287^, ^288^ One of the major challenges, is the fact that majority of commercially available CAD software are predominantly suited for conventional manufacturing processes, such as extrusion, lofts and revolves, and are not capable of generating complex multiscale cellular and lattice structures and specifying multiple material variations within an object.^287^ Therefore, traditional CAD programs cannot generate organic shapes and multiscale geometries, which are required in energy chemistry, for example catalyst support structures. Apart from overcoming of bulk geometric limitations of current CAD programs, a closer link between design and analysis, as well as geometric modelling capabilities are necessary, therefore multi‐physics capabilities that were limited to finite element software will also be needed for CAD programs to harness the geometrical freedom offered by AM.^287^ These novel design strategies for AM need to be developed, in order to resolve the required coupling between geometry, material, analysis, optimization and manufacturing.

### 5.5 Potential for Ultimate Sustainable Manufacturing

**Figure 29:**
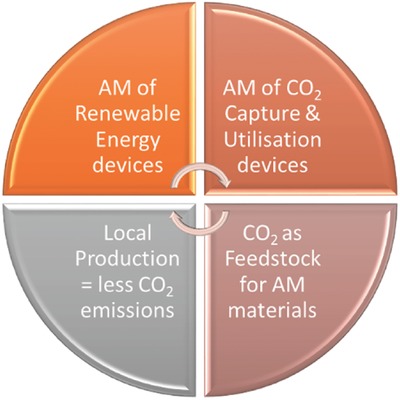
Carbon neutral AM loop.

Recent advances in utilizing AM in energy applications, such as solar energy, electrochemical energy conversion and storage and hydrogen production show an enormous potential of AM as alternative manufacturing technology for renewable energy devices. It is envisioned that AM can also accelerate the progress in developing processes for CO_2_ capture and utilization. It was recently reported that 3D printed portlandite monoliths in form of structural elements such as beam, column or slab can be contacted with captured CO_2_ to form limestone (calcite), which can form dense, monolithic sections that can be used for “green construction”.^289^ Another potential way of mitigating emissions, is to use captured CO_2_ as a feedstock for preparing polymers that can then be used in AM. For instance sustainable polymers can be produced by alternating copolymerization of epoxides and CO_2_ to form polycarbonates.^290^ Reduction of CO_2_ emissions can also result from the potential of AM to disrupt the traditional fossil fuel powered supply chain. Freight transportation of manufactured goods and components amounts to a significant part of global oil consumption. AM is capable of producing fully‐functional devices and parts locally, therefore restricting the freight transportation to just the raw materials. Extensive studies on the full life‐cycle assessment (LCA) of AM technologies are necessary to establish whether AM is less energy intensive than conventional bulk manufacturing process. However, all of the AM technologies use electricity, therefore the electricity generated from renewable energy sources can be used to make it more sustainable. Considering all of these factors, AM has a potential to become an ultimate sustainable manufacturing technology, as illustrated in **Figure** **29**.

## 6 Conclusion

In conclusion, we have communicated the recent progress in additive manufacturing aimed at energy chemistry and material applications. A variety of commercially available AM materials and their properties were summarized and it was proposed that they can be utilized for fabricating structural components of energy devices, depending on the intended application. A number of reports on bespoke energy materials and how they can be additively manufactured with customized solutions were reviewed. The limits of commercially available AM methods in terms of their ability for micro and nanofabrication as well as discoveries on what 3D functional energy structures can be created without design constraints were explored. This review also highlighted emerging and important applications in energy additive manufacturing, including fuel cells, batteries, hydrogen, solar cell as well as carbon capture. The use of AM in microbattery fabrication, shows the most promising potential among several energy applications, as it was demonstrated that AM is capable of fabricating all the essential microbattery components, including 3D electrode configurations and solid‐state electrolytes, which implies that in the near future, it can meet the demand for energy storage microdevices used in portable electronics.

Like any other manufacturing technology, AM suffers from certain limitations. Challenges and obstacles that hinder its use in energy field were discussed for each application, in Section 4. Overall, when compared to subtractive manufacturing, AM has two major limitations: accuracy and material availability. Limitations in achievable accuracy can be expressed in terms of surface quality. Quite often AM fabricated parts show poor surface quality, when compared to smooth surfaces of parts produced by subtractive manufacturing. This stems from the layered nature of AM, where the CAD of the part is sliced into a number of layers, dependent on the layer thickness that was set prior to slicing. As a result the advantage of full automation in AM is achieved by comprising surface quality. In terms of materials selection, since AM is the umbrella term used for various technologies which use different methods to fuse materials together, but generally it can be assumed that the selection of materials in commercially available AM systems is restricted to either polymers, metals and ceramics (although a number of other materials and composites can be processed via DIW). Therefore each AM system is restricted to a particular set of materials. Due to these 2 limitations, AM usually requires additional post‐processing steps (plating, deposition, polishing, etc,) Whereas, subtractive manufacturing is capable of handling any material, with high accuracy, however it requires human intervention and is restricted to parametric features.

What makes AM appealing for energy applications, despite previously mentioned limitations, is the almost complete design freedom offered by a majority of AM technologies. To fully harness the potential of AM in fabrication of energy systems, the development of novel design concepts and strategies, that are inaccessible by conventional manufacturing, is required. One such example, is the previously mentioned topology optimization method, which can be applied to different energy processes, since optimizer can generate intrivcate geometry based on various objective functions and constraints.^47^ Another example, are the nature inspired designs and strategies, where certain features of natural systems can guide the rational design of artificial structures, that use the same fundamental mechanism, which is assisted by theory and experimentation, and should be adapted to the specific context, rather than just mimicry.^39^

In order to advance the application of AM in energy field even further, the developments in AM technologies (nanofabrication and multi‐process AM) should proceed hand‐in‐hand with the development of functional AM materials. As such, AM can be expected in future to become a sustainable manufacturing technology that would compete with traditional subtractive manufacturing.

## Conflict of Interest

The authors declare no conflict of interest.
